# Genomic Diversity and Geographic Distribution of Newcastle Disease Virus Genotypes in Africa: Implications for Diagnosis, Vaccination, and Regional Collaboration

**DOI:** 10.3390/v16050795

**Published:** 2024-05-16

**Authors:** Charlie F. Amoia, Jean N. Hakizimana, Augustino A. Chengula, Muhammad Munir, Gerald Misinzo, James Weger-Lucarelli

**Affiliations:** 1 Department of Veterinary Microbiology, Parasitology and Biotechnology, Sokoine University of Agriculture, P.O. Box 3019, Morogoro 67125, Tanzania; achengula@sua.ac.tz; 2 SACIDS Africa Centre of Excellence for Infectious Diseases, SACIDS Foundation for One Health, Sokoine University of Agriculture, P.O. Box 3297, Morogoro 67125, Tanzania; 3 OR Tambo Africa Research Chair for Viral Epidemics, SACIDS Foundation for One Health, Sokoine University of Agriculture, P.O. Box 3297, Morogoro 67125, Tanzania; hakizimana.jean@sacids.org; 4 Division of Biomedical and Life Sciences, Faculty of Health and Medicine, Lancaster University, Lancaster LA1 4YG, UK; muhammad.munir@lancaster.ac.uk; 5 Department of Biomedical Sciences and Pathobiology, Virginia-Maryland College of Veterinary Medicine, Virginia Tech, Blacksburg, VA 24060, USA

**Keywords:** Newcastle disease, Newcastle disease virus, genotypes, virulence, geographic distribution, phylogeography, Africa

## Abstract

The emergence of new virulent genotypes and the continued genetic drift of Newcastle disease virus (NDV) implies that distinct genotypes of NDV are simultaneously evolving in different geographic locations across the globe, including throughout Africa, where NDV is an important veterinary pathogen. Expanding the genomic diversity of NDV increases the possibility of diagnostic and vaccine failures. In this review, we systematically analyzed the genetic diversity of NDV genotypes in Africa using the Preferred Reporting Items for Systematic Reviews and Meta-Analyses (PRISMA) guidelines. Information published between 1999 and 2022 were used to obtain the genetic background of different genotypes of NDV and their geographic distributions in Africa. The following genotypes were reported in Africa: I, II, III, IV, V, VI, VII, VIII, XI, XIII, XIV, XVII, XVIII, XX, and XXI. A new putative genotype has been detected in the Democratic Republic of the Congo. However, of 54 African countries, only 26 countries regularly report information on NDV outbreaks, suggesting that this number may be vastly underestimated. With eight different genotypes, Nigeria is the country with the greatest genotypic diversity of NDV among African countries. Genotype VII is the most prevalent group of NDV in Africa, which was reported in 15 countries. A phylogeographic analysis of NDV sequences revealed transboundary transmission of the virus in Eastern Africa, Western and Central Africa, and in Southern Africa. A regional and continental collaboration is recommended for improved NDV risk management in Africa.

## 1. Introduction

Newcastle disease virus (NDV) is an important avian pathogen causing severe economic losses in the poultry industry worldwide. NDV is highly contagious and is responsible for severe disease and high mortality rates in susceptible birds [1]. NDV strains can be classified as highly virulent (velogenic), intermediate virulent (mesogenic), or nonvirulent (lentogenic) based on their pathogenicity in birds. Newcastle disease (ND) is caused by *Avian orthoavulavirus 1* (also known as NDV), which belongs to the genus *Orthoavulavirus* of the family *Paramyxoviridae* within the order Mononegavirales [2]. NDV strains are categorized into two main classes (I and II) according to the sequencing of their F genes. Avirulent viruses obtained from wild birds form the majority of class I viruses, which exclusively contain genotype I. Isolates from domestic poultry and wild birds that are both virulent and avirulent fall into class II. There are at least 20 genotypes of class II viruses (I to XXI). The redesigned NDV nomenclature and updated unified phylogenetic categorization scheme do not include genotype XV, which solely consists of recombinant sequences [2]. There are constant reports of newly identified NDV strains from all over the world. Outbreaks of the disease occur periodically as ND is endemic in most African countries [3]. In recent years, there have been several outbreaks of ND in countries with large poultry industries such as Nigeria, Ethiopia, and Egypt [4,5,6,7,8,9]. The outbreaks have been attributed to a range of factors, including inadequate biosecurity measures, poor vaccination coverage, and the movement of infected birds and poultry products.

Poultry farming is a vital source of income and protein for many households in Africa, and ND can cause significant losses in poultry production, leading to food insecurity [10,11]. The emergence of new strains of NDV with increased virulence has further complicated the situation in Africa [12]. These virulent strains are often associated with high mortality rates, leading to significant economic losses for poultry farmers.

The effective control of ND in Africa depends on the consideration of many factors, including disease surveillance, available and accessible reports, and, most importantly, information on the virus strain circulating in poultry populations [13]. There are many strains of ND virus, and the severity of disease caused by each strain can vary [14]. Therefore, knowledge of the NDV strains circulating in an area is essential for the development of effective control strategies, including the selection of appropriate vaccines to be used.

This systematic review aims to identify and examine the genotypic diversity of NDV in Africa. This information will serve as a basis for the development of a genotypic map of NDV in Africa, as a basis to learn more about the level of ND research in Africa, and finally, to gain a better understanding of the virus in order to establish a basis for an ND control strategy in the continent.

## 2. Materials and Methods

### 2.1. Search Strategy

As there was no published information on NDV genotypes present in African countries prior to 1999, following the guidelines set out by the Preferred Reporting Items for Systematic Reviews and Meta-Analyses (PRISMA) [15], a systematic database evaluation of published and publicly available studies in the literature on the occurrence, prevalence, and regional distribution of ND in Africa between 1999 and 2022 was conducted. The following countries and territories were screened for available studies and data: the Democratic Republic of the Congo, Algeria, Angola, Benin, Botswana, Burkina Faso, Burundi, Cameroon, Cape Verde, Central African Republic, Chad, Comoros, Congo, Cote d’Ivoire, Djibouti, Equatorial Guinea, Egypt, Eritrea, Eswatini, Ethiopia, Gabon, Gambia, Ghana, Guinea, Guinea-Bissau, Kenya, the Kingdom of Lesotho, Liberia, Libya, Madagascar, Malawi, Mali, Mauritania, Mauritius, Morocco, Mozambique, Namibia, Niger, Nigeria, Rwanda, Saharawi Arab Democratic Republic, Sao Tome and Principe, Senegal, Seychelles, Sierra Leone, Somalia, South Africa, South Sudan, Sudan, Tanzania, Togo, Tunisia, Uganda, Zimbabwe, and Zambia. The ND outbreak records were retrieved from the World Organization for Animal Health (WOAH), World Animal Health Information System (WAHIS).

### 2.2. Data Extraction and Management

The following online databases were screened for publications: PubMed, Google Scholar, Scopus, CrossRef, Teeal, Hinari, and Agora. The literature search was performed using the search terms (“Newcastle disease” OR “Maladie de Newcastle” OR “Newcastle disease virus” OR “virus de la maladie de Newcastle” OR “ND” OR “NDV” AND (“Algeria” OR “Angola” OR “Benin” OR “Botswana” OR “Burkina Faso” OR “Burundi” OR “Cameroon” OR “Cabo Verde” OR “Central African Republic” OR “Chad” OR “Comoros” OR “Congo” OR “Democratic Republic of the Congo” OR “Cote d’Ivoire” OR “Djibouti” OR “Equatorial Guinea” OR “Egypt” OR “Eritrea” OR “Eswatini” OR “Ethiopia” OR “Gabon” OR “Gambia” OR “Ghana” OR “Guinea” OR “Guinea-Bissau” OR “Kenya” OR “the Kingdom of Lesotho” OR “Liberia” OR “Libya” OR “Madagascar” OR “Malawi” OR “Mali” OR “Mauritania” OR “Mauritius” OR “Morocco” OR “Mozambique” OR “Namibia” OR “Niger” OR “Nigeria” OR “Rwanda” OR “Saharawi Arab Democratic Republic” OR “Sao Tome and Principe” OR “Senegal” OR “Seychelles” OR “Sierra Leone” OR “Somalia” OR “South Africa” OR “South Sudan” OR “Sudan” OR “Tanzania” OR “Togo” OR “Tunisia” OR “Uganda” OR “Zambia” OR “Zimbabwe”). The search was not limited by publication date, and articles in both English and French were included in the review. The “Google Scholar” search engine was used to find papers containing data on the NDV genotype for nations that did not yield any data using the preceding techniques; the search phrase “poultry disease” was used. An account was created on “Rayyan QCRI” [16] and served as a platform for authors to work and exchange information for the preliminary selection of studies. Articles from PubMed, Teeal, Hinari, and Agora were imported directly into Rayyan QCRI. “Publish or Perish” [17] was used to import articles from Google Scholar, Scopus, and CrossRef.

### 2.3. Selection Criteria

This systematic review specifically omitted investigations conducted beyond the confines of Africa or studies failing to document NDV genotypes within the African territory. Additionally, materials such as articles, conference papers, and editorials not written in either English, French, or Portuguese were excluded from consideration. After conducting an initial screening of the abstracts and titles, papers that were deemed potentially relevant were thoroughly examined in their entirety. The criteria used to exclude publications were as follows: (i) only non-ND diseases were reported; (ii) only descriptions of nations and territories outside the approved list were reported; (iii) abstracts only; (iv) descriptions of only ND without the use of sequencing and phylogenetic analysis to determine the NDV genotype; and (v) data that were repeated from another publication. Only review articles with original data that were not readily available elsewhere were considered.

Relevant full-text articles were found using a database search. The following inclusion criteria were used:

- The abstracts of the articles stored on “Rayyan QCRI” were read to ascertain whether they were relevant for inclusion; if necessary, close attention was paid to the introduction, results, and discussion sections of each article to verify their alignment with the inclusion criteria.
- Only articles reporting NDV genotypes in Africa were taken into consideration.

The background information for this study’s discussion of the evolution of NDV genotypes on the African continent came from the chosen studies.

### 2.4. Statistical Analysis

Information was added to a Microsoft Excel database (Microsoft, Redmond, WA, USA). Tables were used to summarize the distribution of reported NDV genotypes by year of isolation, region, and country. A map showing the diversity of NDV genotypes circulating in Africa was generated using the open-source software QGIS version 3.24.1 (https://www.qgis.org/en/site/about/index.html, accessed on 23 June 2023).

### 2.5. Sequence Data Search, Screening, Alignment, and Phylogeographic Analysis

Using the accession number of each strain listed in each of the studies included in the qualitative synthesis, sequence data were acquired from NCBI GenBank. The F, HN, and full genome sequences were screened by filtering out duplicates and incomplete or vaguely annotated sequences. After this sorting process, to facilitate the analysis of the data, a phylogeographic analysis was carried out based on sequences of the NDV F gene. The included sequences were aligned using the ClustalW method implemented in MEGA X [18] to perform genotyping and subsequently edited using SeaView version 5 [19]. The best-fitting nucleotide substitution model was determined using the Akaike information criterion (AIC) as a model selection criterion in the Smart Model Selection in PhyML (SMS) version 1.8.4 [20]. TempEst version 1.5.3 [21] was used to examine the temporal signal and look for outlier sequences. Utilizing BEAST v 1.10.4 along with the BEAGLE library, time-scaled phylogeographic relationships among the Newcastle disease virus (NDV) isolates were established based on the F gene [22]. The phylogeographic analysis was carried out by region, namely Eastern, Western and Central (WC), Northern, and Southern Africa. A GTR + G + I nucleotide substitution model (Gamma+4 distribution) was used to assess evolutionary rate variations and population dynamics within taxa sequences from Southern Africa (SA), the K80 + G nucleotide substitution model (Gamma+4 distribution) was used for Northern Africa (NA), and the K80 + I nucleotide substitution model (Gamma+4 distribution) was used for Eastern Africa (EA) and WC Africa (WCA). The year of collection and the nation where the samples were taken, as indicated in the relevant original report, were marked on the sequencing data. Using Tracer v 1.7.2 [23], the sampling and convergence outcomes of the BEAST model parameters were analyzed, and TreeAnnotator v 1.10.4 [22] was used to annotate the maximum clade credibility (MCC) trees. FigTree v 1.4.4 [23] was used to view and produce annotated trees. With the use of location annotations and the time-scaled tree produced by BEAST, the phylogeography of NDV was rebuilt using PastML, the Felsenstein 1981 (F81) model choices, and maximum likelihood marginal posterior probabilities approximation (MPPA) [24]. The PastML generated tree was visualized and edited using Interactive Tree Of Life (iTOL) v4 [25].

## 3. Results

### 3.1. Search Results and Characteristics of Selected Studies

**Figure 1:**
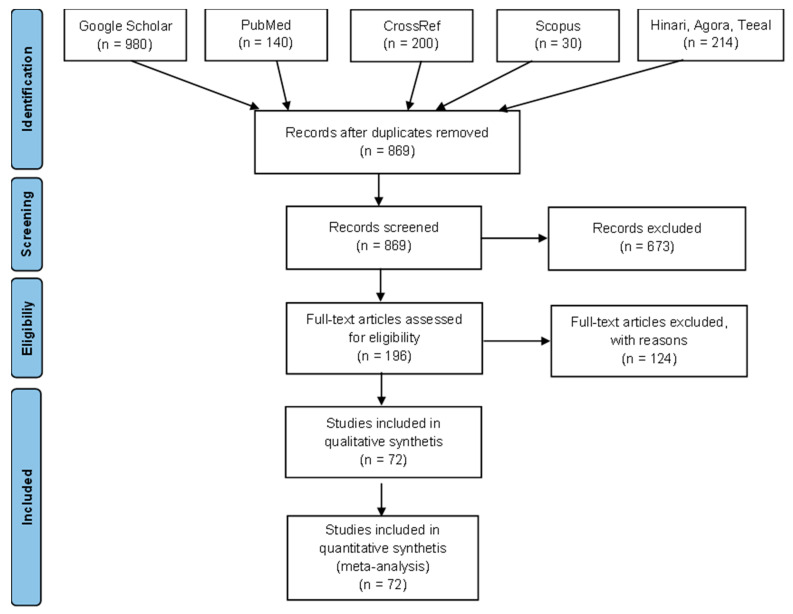
A graphical representation of the search strategy and article selection process until 30 December 2022 illustrated in the PRISMA flowchart.

During the initial search, a total of 1564 articles were gathered, with 869 being retained following the removal of duplicates. The subsequent screening of article titles and abstracts based on the predefined eligibility criteria led to the exclusion of 673 articles. The primary reasons for exclusion were publications reporting data from countries outside the scope of this review, papers lacking genotype identification, and review articles. Consequently, 196 full-text manuscripts underwent a detailed assessment, resulting in the retention of 72 articles for qualitative and quantitative synthesis (Figure 1). Since Herczeg et al.’s [26] work was the first to fulfill the qualifying requirements, the present evaluation takes into account the literature from 1999 to 2022, which is a span of 23 years.

### 3.2. ND Outbreaks

Of the 54 continental and island nations in Africa, only 26 reported data that met our selection criteria: Ethiopia, Tanzania, Uganda, Central African Republic, Togo, Nigeria, Mauritania, Malawi, Sudan, Mozambique, Madagascar, Mali, Benin, Botswana, Burkina Faso, Burundi, Cameroon, Democratic Republic of the Congo, Cote d’Ivoire, Ghana, Kenya, Namibia, Niger, South Africa, Zimbabwe, and Zambia. The total number of ND outbreaks reported to the WOAH by these 26 countries from 2005 to 2022 was 15,970, with 5,503,385 cases that led to 3,475,377 domestic chicken deaths during this period (Table 1). The case fatality rates (CFRs) varied from 1.75% to 100% with an overall CFR of 63.14%. The number of reported ND outbreaks, the number of cases, the number of deaths, and the CFR reported by these 26 countries decreased over time from the 2005–2009 to 2010–2014 periods and increased from the 2010–2014 to 2015–2019 periods. The CFR between 2020 and 2022, which was calculated from data reported by only seven countries, possibly due to the impact of the coronavirus disease 2019 (COVID-19) on disease surveillance in Africa, reaches 92.10% in some cases.

### 3.3. Newcastle Disease Genotype Distribution in Africa

**Figure 2:**
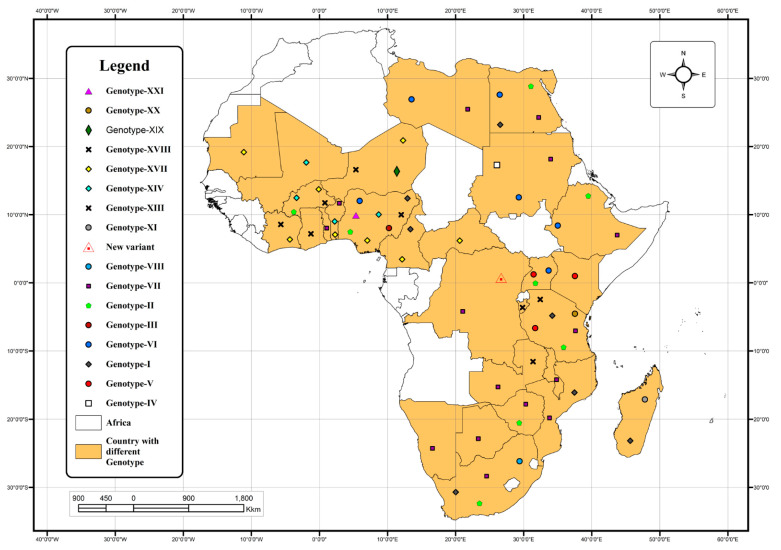
The distribution of NDV genotypes circulating in African countries by December 2022. The map was developed using QGIS version 3.24.1 (https://www.qgis.org/en/site/about/index.html, accessed on 23 June 2023).

The genotypes reported in Africa were I to VIII, X to XVI, XX, and XXI, and a new variant was detected in the Democratic Republic of the Congo [12] (Table 2 and Figure 2).

### 3.4. Eastern Africa

The Eastern Africa region includes the continental countries Sudan, Somalia, Seychelles, Reunion, Kenya, Uganda, South Sudan, Djibouti, Burundi, Ethiopia, Tanzania, Eritrea, Mauritius, Mayotte, Rwanda, and Comoros alongside the island nations of Comoros, Reunion, Mauritius, Seychelles, and Mayotte [27]. However, the available data on NDV genotypes in the region are limited to Burundi, Ethiopia, Kenya, Madagascar, Sudan, Tanzania, and Uganda (Supplementary Table S1).

Within Tanzania, six distinct genotypes of NDV have been characterized, denoted as I, II, V, VII, XIII, and XX [28,29,30,31,32,33]. The two main genotypes reported to be circulating in Kenya are V and VI [34,35,36]. Available data on the status of NDV in Sudan indicates the presence of two genotypes: VI [37] and VI.I [38]. Uganda has experienced resurgent outbreaks with various genotypes, including II, V, and VI [39,40,41]. The available data on the NDV status in Burundi indicates the presence of sub-genotype XIII.1.1 [42]. At least three different genotypes of NDV are circulating in Ethiopia, namely genotypes II, VI, and VII, with a close genetic similarity of virulent isolates with those from Sudan and Egypt [43,44,45,46]. Genotype XI is geographically restricted in Africa and has only been reported from Madagascar, where it circulates between wild birds and domestic chicken [45,47]. Further studies are needed to better understand the ecology and evolution of this sub-genotype and its potential impact on poultry and wild bird populations in the region.

To summarize, NDV is prevalent in Eastern Africa, and several genotypes have been reported in this region. According to recent studies, NDV genotypes II, IV, V, and VII are the most common genotypes circulating in Eastern Africa.

### 3.5. Southern Africa

Southern Africa comprises the following countries: South Africa, Malawi, Eswatini, Angola, Lesotho, Zimbabwe, Zambia, Namibia, Botswana, and Mozambique [48]. In this region, we did not obtain relevant data from three countries: Angola, Eswatini, and Lesotho (Table S2). The extent of Newcastle disease prevalence among village poultry in Mozambique remains inadequately documented. However, two sub-genotypes of genotype VII co-circulate in the country, namely sub-genotypes VII.1.1 and VII.2 [49,50]. Apart from genotypes VII and VIII that appear to be the main genotypes reported in South Africa, other NDV genotypes are still present in South Africa. They include genotypes I to XIV (with VII and VIII inclusive), and their presence is corroborated by previous studies [26,51,52,53,54]. Despite vaccination being widely utilized by farmers in curbing the spread of NDV infection in South Africa, outbreaks continue to be reported [53]. Within Botswana, an examination of samples gathered during Newcastle disease outbreaks in 2014, 2018, and 2019 through a phylogenetic analysis unveiled that all viral strains from Botswana were grouped within sub-genotype VII.2 (formerly classified as subgenus VIIh) and exhibited close genetic affinity with strains originating from South Africa and Mozambique [55]. To prevent the spread of ND in Malawi, various measures are being implemented, including vaccination campaigns, strict biosecurity measures, and the control of bird movements [56]. However, sub-genotype VII.2 was identified in the country in 2020 [49]. The first genetic characterization of NDV isolates in Namibia took place in 2016 on six NDV isolates from backyard poultry. A phylogenetic analysis using the complete F gene sequence revealed that the viruses belonged to a novel subgenotype, VII.2 (VIIk) [57]. In Zambia, the most important factor limiting village poultry production is the high prevalence of diseases, particularly ND [58]. Subgenotype XIII.1.1 and subgenotype VII.2 (VIIh) have been identified to be circulating in the country [49,53]. Zimbabwe is subject to sporadic outbreaks of ND, particularly in extensively raised poultry. Viruses of genotype III have been sporadically isolated in Zimbabwe [59].

In summary, NDV is causing enormous damage in Southern Africa, with the main problem being genotype VII represented by two sub-genotypes, VII.1.1 and VII.2. There is a link between viruses circulating in this region and those in Asia [53].

### 3.6. Western Africa

The United Nations defines Western Africa as the 16 countries of Benin, Burkina Faso, Cape Verde, Gambia, Ghana, Guinea, Guinea-Bissau, Ivory Coast, Liberia, Mali, Mauritania, Niger, Nigeria, Senegal, Sierra Leone, and Togo [60]. However, we did not obtain information for some countries in this region, namely Cape Verde, The Gambia, Guinea, Guinea-Bissau, Liberia, Senegal, and Sierra Leone (Table S3).

In Togo, genotype VII has been found in various studies [61,62]. Nigeria, where several NDV genotypes have been identified, namely genotypes I, II, III, VI, XIV, XVII, XVIII, and XXI, is the country with the highest NDV genotypic diversity in Africa [63,64,65,66]. Genotypes VII, XIV, and XVII are the strains that were already identified in Benin [62,67]. In Burkina Faso, studies have shown that multiple NDV genotypes have been detected, including genotypes II, XVII, and XVII [42,68]. The understanding of Newcastle disease epidemiology in Côte d’Ivoire, along with its current economic ramifications, remains limited. Indeed, genotypes XVII and XVIII co-circulate in Côte d’Ivoire [33,42,67]. In Ghana, NDV genotype XVIII has been identified as responsible for ND outbreaks in the country [29]. Genotype XIV, an NDV variant that originally emerged in West Africa, has been identified as being responsible for ND outbreaks in Mali [69]. Genotype XVII has been identified as being responsible for ND outbreaks in Mauritania [42]. The NDV genotypes co-circulating in Niger are genotypes XIV, XVII, and XVIII [42,63,68].

In conclusion, genotype XVII has been identified as the most geographically dispersed genotype in West Africa, having been found in Nigeria, Côte d’Ivoire, Niger, Burkina Faso, Benin, and Mali. Among the regions studied, West African countries stand out for presenting the most intricate landscape in terms of NDV genetic diversity.

### 3.7. Central Africa

Central Africa consists of the following eight countries: Equatorial Guinea, Chad, São Tomé and Príncipe, Central African Republic, the Democratic Republic of the Congo, Cameroon, Gabon, and Congo-Brazzaville. We only obtained relevant data for our study from three countries in this region, namely Cameroon, the Central African Republic, and the Democratic Republic of the Congo (Table S4).

The available data on the genotypic diversity of NDV in Cameroon show the detection of the genotype XVII strain and strains related to the genotype I vaccine strain (Queensland V4, JX524203) [42,67]. Based on the criteria recently proposed for the classification of NDV strains [2], Central African Republic strains are clustered with genotype XVII [67]. The Democratic Republic of Congo (DRC) has reported the detection of a new variant of NDV and also of subgenotype VII.2 [12].

In summary, Central Africa is characterized by a rather worrying genetic diversity, especially with the presence of new variants. Genotype XVII seems to be the most geographically dispersed genotype, as it is in West Africa.

### 3.8. Northern Africa

Northern Africa consists of seven countries: Tunisia, Morocco, Western Sahara, Algeria, Egypt, and Libya. However, we only obtained relevant data from two countries, namely Libya and Egypt (Table S5).

Two genotypes characterized by their large number of sub-genotypes have been observed in Libya, namely genotypes VI [70] and VII [71]. Currently, there are at least two NDV genotypes circulating in chickens in Egypt: II and VII [72,73,74,75,76,77].

To summarize, genotype VII, represented by its subgenotype VII.1.1, is the predominant genotype circulating in this region of Africa as it has been associated with numerous outbreaks of ND in chicken.

### 3.9. Phylogenetic and Phylogeographic Analyses

For enhanced comprehension of the evolutionary trajectory and spatiotemporal fluctuations of NDV in Africa, a phylogeographic investigation focusing on prevalent NDV genotypes was undertaken. In total, 847 NDV nucleotide sequences were downloaded from the National Center for Biotechnology Information (NCBI) GenBank database comprising NDV strains from 27 African countries, namely Benin (n = 26), Burkina Faso (n = 13), Botswana (n = 14), Burundi (n = 2), Cameroon (n = 3), the Central African Republic (n = 3) Côte d’Ivoire (n = 8), the Democratic Republic of the Congo (n = 3), Egypt (n = 141), Ethiopia (n =43), Ghana (n = 4), Kenya (n = 57), Libya (n = 4), Madagascar (n =16), Mali (n = 7), Mauritania (n = 1), Mozambique (n = 15), Namibia (n =6), Niger (n = 22), Nigeria (n = 190), South Africa (n = 77), Sudan (n = 10), Tanzania (n = 81), Togo (n = 6), Uganda (n = 89), Zambia (n = 4), and Zimbabwe (n = 2). No sequences were found from other African countries. All NDV sequences used in this study were sampled in Africa from 1968 to 2022. After this sorting process, to facilitate the analysis of the data, a phylogeographic analysis was carried out based on sequences of the NDV F gene. The resulting alignment consisted of 313 sequences, including 121 from Eastern Africa, 76 from Northern Africa, 42 from Southern Africa, and 74 from Western and Central Africa. A Bayesian statistical framework was employed to estimate the evolutionary history of the 313 F gene sequences collected in Africa from 1968 to 2022 to infer viral diffusion between geographic regions.

**Figure 3:**
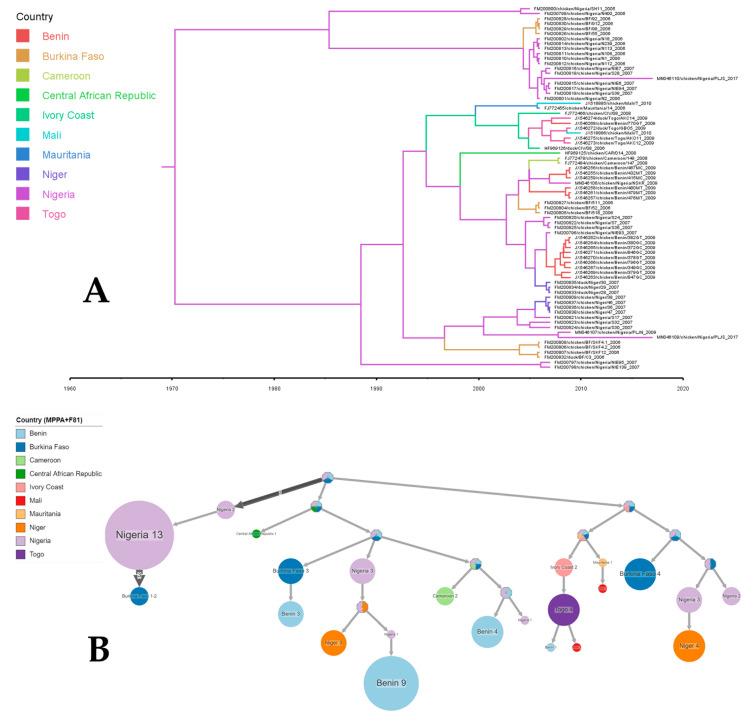
The ancestral reconstruction of Newcastle disease virus strains collected in Western and Central Africa. The figure shows the full tree (**A**) produced using FigTree v 1.4.4 after a phylogeographic analysis using BEAST and compressed (**B**) visualizations produced by PastML using MPPA with an F81-like model.

For Western and Central Africa: The outcomes of the root-to-tip divergence analysis demonstrated that the dataset employed in this research exhibited a positive temporal signal, as indicated by a correlation coefficient of 0.17 and an R-squared value of 0.029. The time to the most recent common ancestor (TMRCA) for NDV strains circulating in the Western and Central Africa regions dated back to 1968 (95% CI of 1857–1989) with an evolutionary rate of 3.129 × 10^−3^ (9.9185 × 10^−4^, 6.0981 × 10^−3^). The precise location of the root remained unresolved, although there was an indication pointing towards Nigeria as a potential root location with a probability of 53.90% (Figure 3A).

An examination of the phylogeographic dispersal patterns of NDV identified several instances of cross-border spread, notably originating from Nigeria and extending into Niger, Burkina Faso, and Benin. From Cote d’Ivoire, NDV further spread to Togo, Benin, and Mali. Transmissions from West Africa to Cameroon, the Central African Republic, and DRC were also observed (Figure 3B).

For Eastern Africa: An examination of root-to-tip divergence unveiled a positive temporal signal within the dataset utilized in this study, as evidenced by a correlation coefficient of 0.69 and an R-squared value of 0.48.

**Figure 4:**
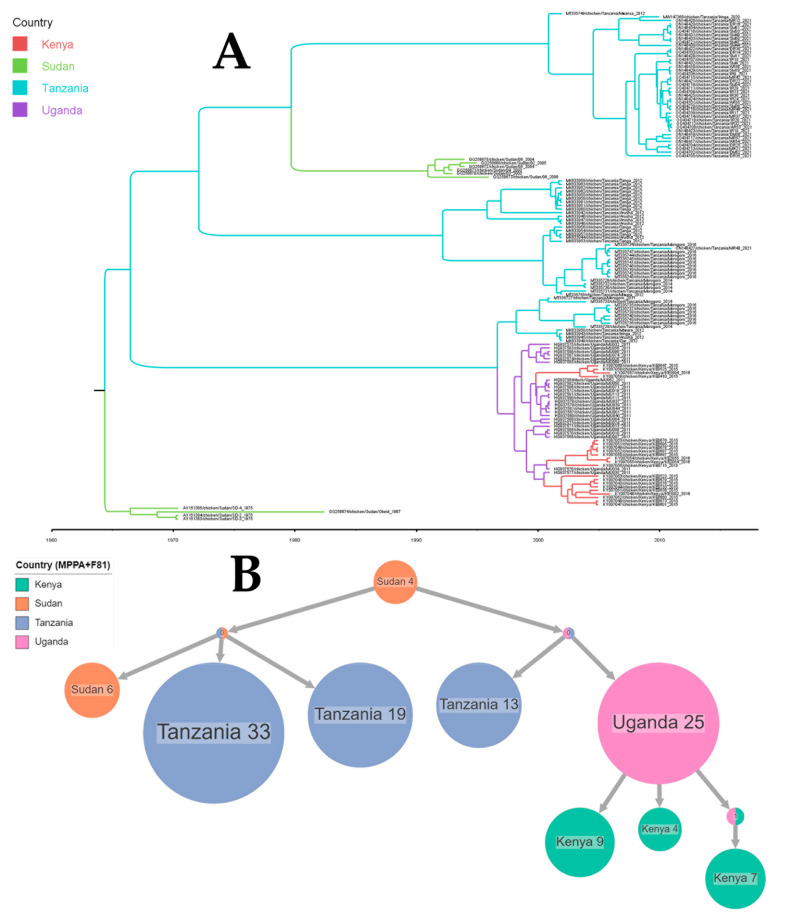
An ancestral reconstruction of Newcastle disease virus strains collected in Eastern Africa. The figure shows the full tree (**A**) produced using FigTree v 1.4.4 after a phylogeographic analysis using BEAST and compressed (**B**) visualizations produced with PastML using MPPA with an F81-like model.

The TMRCA for NDV strains circulating in the Eastern Africa region dated back to 1965 (95% CI: 1905–1974) with an evolutionary rate of 2.565 × 10^−3^ (1.4121 × 10^−3^, 4.3197 × 10^−3^). The determination of the root’s location revealed Sudan as a potential root location, suggested with a probability of 71.25% (Figure 4A). A time-scaled phylogeographic analysis suggested Sudan and Uganda as potential sources of the Tanzanian NDV strains. Transmissions from Uganda to Kenya and Tanzania and from Tanzania to Uganda and Sudan were also observed (Figure 4B).

For Southern Africa: An examination of root-to-tip divergence revealed a positive temporal signal within the dataset utilized in this study, characterized by a correlation coefficient of 0.67 and an R-squared value of 0.45. The TMRCA for NDV strains circulating in the Southern Africa region dated back to 1977 (95% CI: 1931–1996) with an evolutionary rate of 1.596 × 10^−3^ (7.9621 × 10^−4^, 2.7545 × 10^−3^).

**Figure 5:**
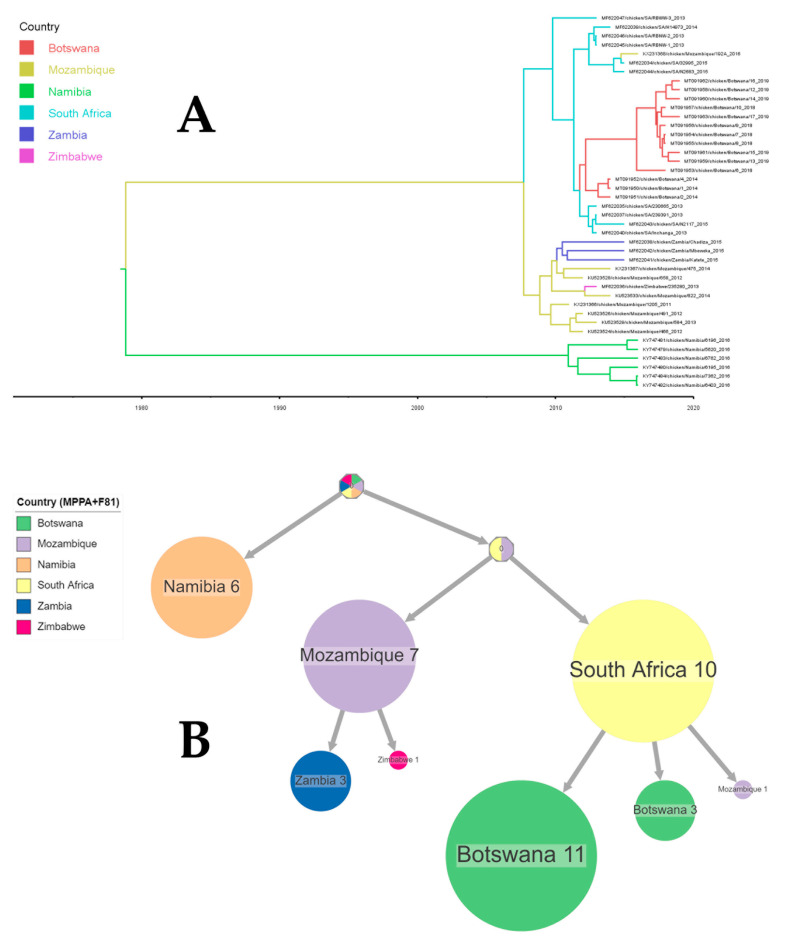
An ancestral reconstruction of Newcastle disease virus strains collected in Southern Africa. The figure shows the full tree (**A**) produced using FigTree v 1.4.4 after a phylogeographic analysis using BEAST and compressed (**B**) visualizations produced with PastML using MPPA with an F81-like model.

In Figure 5, a phylogeographic analysis of the fusion gene’s full-length nucleotide sequence is depicted, specifically exploring the 42 Southern African sub-genotypes, including VII.2 NDV. Despite this, the root’s location remained inconclusive, with an indication suggesting Namibia as a possible root location with a probability of 41.07% (Figure 5A). Furthermore, a time-scaled phylogeographic analysis utilizing F gene nucleotide sequences proposed South Africa as a potential origin for the NDV strains found in Mozambique and Botswana. Transmissions from Mozambique to Zambia and Zimbabwe were also observed (Figure 5B).

For Northern Africa: An analysis of root-to-tip divergence unveiled that the dataset from North Africa (Egypt and Libya) utilized in this study portrayed a negative temporal signal characterized by a correlation coefficient of −0.2313. Thus, a phylogeographic analysis could not be carried out for North Africa due to insufficient data.

## 4. Limitation

The main limitation of our analysis lies in the fact that we have relied solely on publicly accessible data. Assessing underreporting bias presents a quantification challenge. Notably, several African nations encountering recent ND outbreaks are omitted from this genotyping analysis due to a lack of information on the NDV genotypes that caused these various outbreaks (Table 1). These African countries could therefore play a very important role in the spread of NDV on the continent, if not elsewhere. It is also possible that NDV strains from Africa available on Genbank have not been used in publications, and that more genotypes are present in some African countries. Notwithstanding these constraints, the sequences utilized in our analyses constitute the most comprehensive dataset on NDV that is available, encompassing information gathered from across the entirety of Africa. Continued monitoring, sampling, and a molecular analysis of the data, particularly in poorly represented areas, would improve future analyses.

## 5. Discussion

This systematic review reports the geographical distribution of different NDV genotypes in Africa. Of the 54 continental and island nations in Africa, only 26 reported ND information to WOAH. Our results show that NDV is endemic in at least 26 African countries with repeated outbreaks in most of these countries. The number of reported ND outbreaks, cases, deaths, and case fatality rates for these 26 countries have been increasing since 2010 despite the increased practice of vaccination by African farmers, as reported by several authors [78,79,80]. The results of this study clearly confirm that virus dispersal was not only regional, in that several countries in different parts of Africa were shown to have several genotypes and sub-genotypes in common. This was previously demonstrated by Fentie et al. [46], who provided evidence of at least three different genotypes of NDV circulating in Ethiopian (East Africa) rural backyards, namely genotypes II, VI, and VII in close genetic similarity to virulent isolates from Sudan (North Africa) and Egypt (North Africa). This is further evidence of potential epidemiological links between NDV outbreaks in different African countries, probably due to geographical proximity and cross-border poultry movements.

This study also provides an update on the history of NDV genotypes that were already identified in Africa, namely I, II, III, IV, V, VI, VII, VIII, XI, XIII, XIV, XVII, XVIII, XX, and XXI, and a new variant detected in the Democratic Republic of the Congo. That is a total of 16 out of the 21 existing genotypes based on the current nomenclature [2]. The restricted distribution of genotype XI in Madagascar suggests that it may have unique genetic characteristics and transmission dynamics compared to other NDV subtypes. The most prevalent genotype in Africa is genotype VII (reported in 15 African countries), followed by genotypes II (8 countries) and XVII (8 countries).

The main NDV strains currently used for the production of vaccines against ND and marketed in Africa are LaSota (lentogenic), I-2 (avirulent), Mukteswar (avirulent), Komarov (mesogenic), Hitchner B1 (lentogenic), V4 (avirulent), and V4-HR (avirulent) [33]. The most commonly used vaccine strains are LaSota, Hitchner B1, and I2, which remain the precursors for the majority of vaccines that are currently available on the market. The coverage and frequency of ND vaccination vary considerably between regions and countries. In Tanzania (East Africa), the most commonly used vaccines are LaSota and I-2, in addition to V4-HR [81,82]. However, in Mali (West Africa), it has been observed that, in addition to LaSota and I-2, Hitchner B1 is the most commonly used vaccine by many farmers. It was previously thought that these conventional commercial NDV vaccines were effective against NDV. However, it has been demonstrated that they are not able to entirely prevent infection and virus shedding due to genotype differences between the vaccine and circulating viruses [83,84]. The LaSota vaccine offers limited protection against certain strains of NDV, as evidenced by the results of cross-protection tests conducted [85,86]. Since genotype VII is the most prevalent group of NDV in Africa, which was reported in 15 countries, the development of a recombinant vaccine from genotype VII seems, more than ever, a solution to be considered for a more efficient fight against ND in Africa.

Despite the inherent biases and constraints within the genetic data concerning NDV at the African level, molecular epidemiological analyses utilizing self-submitted genetic data have proven effective in examining the dynamics and ecology of viral transmission. Similar models leveraging self-submitted genetic data have been employed in the study of viral pathogens, such as avian influenza virus [81,82], Ebola virus [83], African swine fever virus [84], foot-and-mouth disease virus [85], and NDV [34,86,87]. Using a similar approach through a Bayesian phylogeographical analysis, we were able to provide useful information for future surveillance efforts by presenting measurable epidemiological links. Ultimately, our results emphasize the critical need for expanded sampling and sequencing efforts concerning NDV isolates throughout Africa. These endeavors are essential for refining estimations of virus migration, thus enhancing our understanding and the potential mitigation of viral movement.

Further research is needed to improve our understanding of the factors involved in the spread and maintenance of the virus in the different regions of Africa and the development of a regional approach to mitigate the spread of NDV. The results of this study highlight the ongoing need for field surveillance for notifiable viruses. Given that most countries in sub-Saharan Africa face several challenges, including data quality and ineffective early warning systems [88], there is a dire need to build the capacity of stakeholders (consumers, livestock keepers, veterinarians, and government officials) in data management and information sharing.

## 6. Conclusions

This study provides an overview of the spatial and temporal trends of ND in different regions of Africa. Overall, it can be concluded that ND remains endemic in Africa with strong viral genotypic diversity. Based on the findings of this review, it is imperative to promptly enact suitable action plans for more research on NDV in every African country, followed by cooperation and information sharing between countries at the regional and sub-regional levels.

## Figures and Tables

**Table 1:** Reports of ND outbreaks, cases, deaths, and the corresponding case fatality rate submitted to the World Organization for Animal Health (WOAH) by some African countries in the 2005–2022 period.

Period		Subregion	Country	Number of Outbreaks	Number of Cases	Number of Deaths	Case Fatality Rate (%)
			Benin	214	67,763	36,523	53.90
			Burkina Faso	175	17,887	10,474	58.56
			Ivory Coast	14	80,400	28,117	34.97
2005–2009		Western Africa	Ghana	468	102,937	29,485	28.64
			Mali	19	6279	5428	86.45
			Mauritania	10	1556	1051	67.54
			Niger	1	220	210	95.45
			Nigeria	97	29,771	8049	27.04
			Togo	269	19,370	11,804	60.94
			Cameroon	155	27,184	15,305	56.30
		Central Africa	CAR *	5	589	456	77.42
			DRC *				
			Burundi				
			Ethiopia	212	22,574	15,194	67.31
			Kenya	70	71	66	92.96
		Eastern Africa	Madagascar	46	3924	2177	55.48
			Sudan	9	33,081	835	2.52
			Tanzania	323	58,378	48,301	82.74
			Uganda	10	32,321	4903	15.17
			Botswana	30	3145	2680	85.21
			Malawi	7	16	13	81.25
			Mozambique	28	5181	3656	70.57
		Southern Africa	Namibia	20	599	446	74.46
			South Africa	447	1,785,647	1,395,053	78.13
			Zambia	417	96,281	30,499	31.68
			Zimbabwe	135	30,700	14,257	46.44
	Subtotal			3181	2,425,874	1,664,982	68.63
			Benin	481	79,254	37,974	47.91
			Burkina Faso	294	45,539	21,447	47.10
			Ivory Coast	49	36,283	17,196	47.39
2010–2014		Western Africa	Ghana	830	147,448	36,551	24.79
			Mali	14	27,134	21,443	79.03
			Mauritania	4	53	51	96.23
			Niger	31	2540	2114	83.23
			Nigeria	160	35,650	12,231	34.31
			Togo	115	9444	5948	62.98
			Cameroon	64	11,036	4316	39.11
		Central Africa	CAR *	25	1819	1415	77.79
			DRC *	72	319,511	275,024	86.08
			Burundi	86	15,148	459	3.03
			Ethiopia	409	37,574	14,750	39.26
			Kenya	37	2267	1129	49.80
		Eastern Africa	Madagascar	47	896	896	100
			Sudan	15	15,579	12,157	78.03
			Tanzania	77	13,783	10,720	77.78
			Uganda				
			Botswana	44	10,140	3477	34.29
			Malawi	23	10,490	10,295	98.14
			Mozambique	19	38,998	15,007	38.48
		Southern Africa	Namibia	10	367	317	86.38
			South Africa	217	840,367	526,102	62.60
			Zambia	563	126,380	63,611	50.33
			Zimbabwe	113	34,776	19,655	56.52
	Subtotal			3799	1,862,476	1,114,285	59.83
			Benin	353	26,717	11,345	42.46
			Burkina Faso	295	40,025	17,064	42.63
			Ivory Coast	67	52,623	26,843	51.01
2015–2019		Western Africa	Ghana	928	146,059	75,973	52.02
			Mali	10	35,892	26,426	73.63
			Mauritania				
			Niger	267	3978	1779	44.72
			Nigeria	44	6231	502	8.06
			Togo	118	5417	3093	57.10
			Cameroon	72	17,469	12,814	73.35
		Central Africa	CAR *	46	1566	801	51.15
			DRC *	107	127,430	126,909	99.59
			Burundi	197	90,928	1587	1.75
			Ethiopia	73	16,295	8707	53.43
			Kenya	85	6796	3287	48.37
		Eastern Africa	Madagascar	93	7824	3037	38.82
			Sudan	31	23,747	21,534	90.68
			Tanzania	46	4953	3254	65.70
			Uganda				
			Botswana	136	1280	1107	86.48
			Malawi	6	16,404	16,037	97.76
			Mozambique	14	2620	1872	71.45
		Southern Africa	Namibia	66	3347	2997	89.54
			South Africa	160	39,396	33,346	84.64
			Zambia	761	163,856	96,171	58.69
			Zimbabwe	656	76,254	73,696	96.65
	Subtotal			4631	917,107	570,181	62.17
			Benin				
			Burkina Faso				
			Ivory Coast	5	3966	2237	56.40
2020–2022		Western Africa	Ghana				
			Mali				
			Mauritania				
			Niger				
			Nigeria	185	89,016	9977	11.21
			Togo	1	563	235	41.74
			Cameroon				
		Central Africa	CAR *				
			DRC *				
			Burundi				
			Ethiopia	163	85,511	43,031	50.32
			Kenya	45	715	438	61.25
		Eastern Africa	Madagascar				
			Sudan				
			Tanzania				
			Uganda				
			Botswana				
			Malawi				
			Mozambique				
		Southern Africa	Namibia				
			South Africa	29	4371	4026	92.10
			Zambia	133	113,786	65,985	57.99
			Zimbabwe				
	Subtotal			4359	297,928	125,929	42.26
	Grand total			15,970	5,503,385	3,475,377	63.14

* DRC = Democratic Republic of the Congo. * CAR = Central African Republic.

**Table 2:** A summary of the NDV genotype distribution in 28 African countries. The black highlight indicates that the corresponding genotype is present.

Subregion	Country	NDV Genotypes																					Total Genotypes
		I	II	III	IV	V	VI	VII	VIII	IX	X	XI	XII	XIII	XIV	XVI	XVII	XVIII	XIX	XX	XXI	NV ^a^	
	Benin																						3
	Burkina Faso																						4
	Ivory Coast																						2
Western Africa	Ghana																						1
	Mali																						1
	Mauritania																						1
	Niger																						3
	Nigeria																						8
	Togo																						1
	Cameroon																						2
Central Africa	CAR *																						1
	DRC *																						2
	Burundi																						1
	Ethiopia																						3
	Kenya																						1
Eastern Africa	Madagascar																						2
	Sudan																						3
	Tanzania																						6
	Uganda																						3
	Botswana																						1
	Malawi																						1
	Mozambique																						2
Southern Africa	Namibia																						1
	South Africa																						4
	Zambia																						2
	Zimbabwe																						2
Northern Africa	Egypt																						4
	Libya																						2
Total Countries affected		6	8	1	1	3	6	15	1	0	0	0	0	3	5	0	8	5	0	1	1	1	

^a^: New variant. * DRC = Democratic Republic of the Congo. * CAR = Central African Republic.

## Data Availability

The datasets used and/or analyzed during the current study are available from the corresponding author upon reasonable request.

## Supplementary Materials

The following supporting information can be downloaded at https://www.mdpi.com/article/10.3390/v16050795/s1, Table S1: Distribution of class II NDV genotypes in Eastern Africa, Table S2: Distribution of class II NDV genotypes in Southern Africa, Table S3: Distribution of class II NDV genotypes in Western Africa, Table S4: Distribution of class II NDV genotypes in Central Africa, Table S5: Distribution of class II NDV genotypes in Northern Africa.

## Abbreviations

ND: Newcastle disease; NDV: Newcastle disease virus; CFR: case fatality rates; WOAH: World Organization for Animal Health; PRISMA: Preferred Reporting Items for Systematic Reviews and Meta-Analyses; WAHIS: World Animal Health Information System; EA: Eastern Africa; SA: Southern Africa; WC: Western and Central; NA: Northern Africa; TMRCA: time to the most recent common ancestor.
