# Supplementary material for: Genomic Diversity and Geographic Distribution of Newcastle Disease Virus Genotypes in Africa: Implications for Diagnosis, Vaccination, and Regional Collaboration
Source: Viruses. 2024 May 16;16(5):795. doi: 10.3390/v16050795 (PMC11125703; doi:10.3390/v16050795)
Supplement: Supplementary file 1 [file viruses-16-00795-s001.zip › Table S1 Distribution of class II NDV genotypes in Eastern Africa.pdf]

Table S1: Distribution of class II NDV genotypes in Eastern Africa

Country	Paper	Isolate GenBank accession number	Genotype	Year of collection	Country	Isolate name	Reference
**Tanzania**	**1**	AY 175653	V	1995	Tanzania	AV 1300/95 MG-10-04-CTanzania	(Aldous *et al.*, 2003)
		AY 175654	V	1995	Tanzania	AV 1300/95 MG-10-05-CTanzania	
		AY 175655	XX	1995	Tanzania	AV 1300/95 TB-02-13-GTanzania	
		AY 175656	V	1995	Tanzania	AV 1300/95 TB-02-24-DTanzania	
		AY 175728	V	1995	Tanzania	AV 1300/95 MG-04-08CTanzania	
	**2**	MT335727	V	2011	Tanzania	VirMagerHatch/chicken/Morogoro/Tanzania/2011	(da Silva *et al.*, 2020)
		MT335728	V	2014	Tanzania	9583/chicken/Morogoro/Tanzania/2014	
		MT335729	XIII1.1	2014	Tanzania	6094/chicken/Morogoro/Tanzania/2014	
		MT335730	XIII1.1	2014	Tanzania	9600/chicken/Morogoro/Tanzania/2014	
		MT335731	XIII1.1	2014	Tanzania	9676/chicken/Morogoro/Tanzania/2014	
		MT335732	XIII1.1	2014	Tanzania	9581/chicken/Morogoro/Tanzania/2014	
		MT335733	V	2014	Tanzania	30Hatch/chicken/Morogoro/Tanzania/2014	
		MT335734	XIII1.1	2016	Tanzania	9206/chicken/Morogoro/Tanzania/2016	
		MT335735	V	2016	Tanzania	208/chicken/Morogoro/Tanzania/2016	
		MT335736	V	2016	Tanzania	6467/chicken/Morogoro/Tanzania/2016	

		MT335737	V	2016	Tanzania	9455/chicken/Morogoro/Tanzania/2016	
		MT335738	XIII1.1	2016	Tanzania	9261/chicken/Morogoro/Tanzania/2016	
		MT335739	XIII1.1	2016	Tanzania	8037/chicken/Morogoro/Tanzania/2016	
		MT335740	V	2016	Tanzania	9443/chicken/Morogoro/Tanzania/2016	
		MT335741	XIII1.1	2016	Tanzania	189/chicken/Morogoro/Tanzania/2016	
		MT335742	XIII1.1	2016	Tanzania	525/chicken/Morogoro/Tanzania/2016	
		MT335743	V	2016	Tanzania	320-2719/chicken/Morogoro/Tanzania/2016	
		MT335744	XIII1.1	2016	Tanzania	9334/chicken/Morogoro/Tanzania/2016	
		MT335745	XIII1.1	2016	Tanzania	8659/chicken/Morogoro/Tanzania/2016	
		MT335746	XIII1.1	2016	Tanzania	9381-131/chicken/Morogoro/Tanzania/2016	
		MT335747	XIII1.1	2017	Tanzania	10Vac/chicken/Morogoro/Tanzania/2017	
		MT335748	XIII1.1	2017	Tanzania	11Vac/chicken/Morogoro/Tanzania/2017	
		MT335749	VII.2	2012	Tanzania	Mw13/chicken/Mwanza/Tanzania/2012	
		MT335750	XIII1.1	2012	Tanzania	Mt07/chicken/Mtwara/Tanzania/2012	
	**3**	MK583011	V	2012	Tanzania	chicken/Tanzania/Mbeya/ MT15/2012	(Goraichuk *et al.*, 2019)
	**4**	MW147368	VII	2020	Tanzania	NDV/Chicken/Tanzania/Iringa/2020	(Kibasa, 2020)
	**5**	MK633932	XIII 1.1	2012	Tanzania	APMV-1/Chicken/Tanzania-Mbeya/MS1/2012	(Msoffe *et al.*, 2019)

		MK633934	XIII 1.1	2012	Tanzania	APMV-1/chicken/Tanzania-Iringa/IC20/2012	
		MK633935	XIII 1.1	2012	Tanzania	APMV-1/chicken/Tanzania-Iringa/IM40/2012	
		MK633937	XIII 1.1	2012	Tanzania	APMV-1/chicken/Tanzania-Arusha/AC31/2012	
		MK633945	V	2012	Tanzania	APMV-1/chicken/Tanzania-Arusha/AC56/2012	
		MK633941	XIII 1.1	2012	Tanzania	APMV-1/chicken/Tanzania-Tanga/N71/2012	
		MK633938	XIII 1.1	2012	Tanzania	APMV-1/chicken/Tanzania-Arusha/AK5/2012	
		MK633936	XIII 1.1	2012	Tanzania	APMV-1/chicken/Tanzania-Arusha/AC11/2012	
		MK633942	XIII 1.1	2012	Tanzania	APMV-1/chicken/Tanzania-Arusha/AC12/2012	
		MK633939	XIII 1.1	2012	Tanzania	APMV-1/chicken/Tanzania-Arusha/AC41/2012	
		MK633940	XIII 1.1	2012	Tanzania	APMV-1/chicken/Tanzania-Arusha/AC42/2012	
		MK633944	XIII 1.1	2012	Tanzania	APMV-1/chicken/Tanzania-Arusha/AC53/2012	
		MK633946	XIII 1.1	2012	Tanzania	APMV-1/chicken/Tanzania-Arusha/AK3/2012	
		MK633947	XIII 1.1	2012	Tanzania	APMV-1/chicken/Tanzania-Arusha/AK8/2012	
		MK633948	XIII 1.1	2012	Tanzania	APMV-1/chicken/Tanzania-Arusha/A18/2012	
		MK633933	XIII 1.1	2012	Tanzania	APMV-1/chicken/Tanzania-Mbeya/MT1/2012	
		MK633951	XIII 1.1	2012	Tanzania	APMV-1/chicken/Tanzania-Tanga/N1/2012	
		MK633952	XIII 1.1	2012	Tanzania	APMV-1/chicken/Tanzania-Tanga/N6/2012	

		MK633953	XIII 1.1	2012	Tanzania	APMV-1/chicken/Tanzania-Tanga/N34/2012	
		MK633954	XIII 1.1	2012	Tanzania	APMV-1/chicken/Tanzania-Tanga/N38/2012	
		MK633955	XIII 1.1	2012	Tanzania	APMV-1/chicken/Tanzania-Tanga/N41/2012	
		MK633956	XIII 1.1	2012	Tanzania	APMV-1/chicken/Tanzania-Tanga/N47/2012	
		MK633957	XIII 1.1	2012	Tanzania	APMV-1/chicken/Tanzania-Tanga/N49/2012	
		MK633958	XIII 1.1	2012	Tanzania	APMV-1/chicken/Tanzania-Tanga/N63/2012	
		MK633959	XIII 1.1	2012	Tanzania	APMV-1/chicken/Tanzania-Tanga/N76/2012	
		MK633960	XIII 1.1	2012	Tanzania	APMV-1/chicken/Tanzania-Tanga/N78/2012	
		MK633961	XIII 1.1	2012	Tanzania	APMV-1/chicken/Tanzania-Tanga/N80/2012	
		MK633962	XIII 1.1	2012	Tanzania	APMV-1/chicken/Tanzania-Tanga/N85/2012	
		MK633963	XIII 1.1	2012	Tanzania	APMV-1/chicken/Tanzania-Tanga/N91/2012	
		MK633949	V	2012	Tanzania	APMV-1/chicken/Tanzania-Dar es Salaam/B280/2012	
		MK633950	V	2012	Tanzania	APMV-1/chicken/Tanzania-Dar es Salaam/K8/2012	
		MK633943	V	2012	Tanzania	APMV-1/chicken/Tanzania-Iringa/IM4/2012	
	**6**	AY160131	I	1995	Tanzania	MG6.11C	(Yongolo *et al.*, 2011)
		AY160152	I	1995	Tanzania	MG7.10C	
		AY160149	II	1995	Tanzania	MB9.38C	

		FJ713136	II	1995	Tanzania	TB9.18C	
		AY160129	II	1995	Tanzania	MG10.3C	
		AY160145	II	1995	Tanzania	MG1.15C	
		AY160140	XX	1995	Tanzania	MG3.35C	
		AY160154	XX	1995	Tanzania	MG6.30C	
		AY160135	XX	1995	Tanzania	MG6.16C	
		AY160142	XX	1995	Tanzania	MG6.9C	
		AY160141	V	1995	Tanzania	MG6.28C	
		AY160134	V	1995	Tanzania	MG2.40C	
		AY160130	V	1995	Tanzania	MG2.32C	
		AY160139	V	1995	Tanzania	MG10.5C	
**Kenya**	**1**	KY007043	V	2015	Kenya	NDV/chicken/Kenya/KE0576/2015	(Ogali *et al.*, 2018)
		KY007044	V	2015	Kenya	NDV/chicken/Kenya/KE0733/2015	
		KY007045	V	2015	Kenya	NDV/chicken/Kenya/KE0679/2015	
		KY007046	V	2015	Kenya	NDV/chicken/Kenya/KE0673/2015	
		KY007047	V	2015	Kenya	NDV/chicken/Kenya/KE0601/2015	
		KY007048	V	2016	Kenya	NDV/chicken/Kenya/KE1002/2016	
		KY007049	V	2015	Kenya	NDV/chicken/Kenya/KE0678/2015	
		KY007050	V	2015	Kenya	NDV/chicken/Kenya/KE0697/2015	
		KY007051	V	2015	Kenya	NDV/chicken/Kenya/KE0695/2015	
		KY007052	V	2015	Kenya	NDV/chicken/Kenya/KE0687/2015	

		KY007053	V	2015	Kenya	NDV/chicken/Kenya/KE0676/2015	
		KY007054	V	2016	Kenya	NDV/chicken/Kenya/KE2055/2016	
		KY007055	V	2016	Kenya	NDV/chicken/Kenya/KE2054/2016	
		KY007056	V	2015	Kenya	NDV/chicken/Kenya/KE0715/2015	
		KY007057	V	2016	Kenya	NDV/chicken/Kenya/KE1004/2016	
		KY007058	V	2015	Kenya	NDV/chicken/Kenya/KE0525/2015	
		KY007059	V	2015	Kenya	NDV/chicken/Kenya/KE0410/2015	
		KY007060	V	2015	Kenya	NDV/chicken/Kenya/KE0647/2015	
		KY007061	V	2015	Kenya	NDV/chicken/Kenya/KE0638/2015	
		KY007062	V	2015	Kenya	NDV/chicken/Kenya/KE0660/2015	
		KY007063	V	2016	Kenya	NDV/chicken/Kenya/KE0523/2015	
	**2**	MN685354	V	2015	Kenya	Kenya/chicken/Makueni/KE001/2015	(Ogali *et al.*, 2020)
		MN685355	V	2016	Kenya	Kenya/chicken/Mombasa/KE0811/2016	
		MN685356	V	2015	Kenya	Kenya/chicken/Malaba/KE0698/2015	
	**3**	MW342776	V.3	2017	Kenya	MW342776/ck/KE/Nairobi/Kariobangi_North/A376/2017/(V.3)	(Kariithi *et al.*, 2021)
		MW342777	V.3	2017	Kenya	MW342777/ck/KE/Nairobi/Kariobangi_North/A371/2017/(V.3)	
		MW342778	V.3	2017	Kenya	MW342778/ck/KE/Nairobi/Kariobangi_North/A374/2017/(V.3)	
		MW342779	V.3	2017	Kenya	MW342779/ck/KE/Nairobi/Kariobangi_North/A373/2017/(V.3)	
		MW342780	V.3	2018	Kenya	MW342780/ck/KE/Kilifi/Sokoni/3FB28/2018/(V.3)	
		MW342781	V.3	2018	Kenya	MW342781/ck/KE/Kilifi/Sokoni/3FB35/2018/(V.3)	
		MW342782	V.3	2018	Kenya	MW342782/ck/KE/Kilifi/Sokoni/3FB31/2018/(V.3)	
		MW342783	V.3	2018	Kenya	MW342783/ck/KE/Kilifi/Sokoni/3FB33/2018/(V.3)	

		MW342784	V.3	2018	Kenya	MW342784/ck/KE/Kilifi/Sokoni/3FB26/2018/(V.3)	
		MW342785	V.3	2018	Kenya	MW342785/ck/KE/Kilifi/Sokoni/3FB32/2018/(V.3)	
		MW342786	V.3	2018	Kenya	MW342786/ck/KE/Kilifi/Sokoni/3FB34/2018/(V.3)	
		MW342787	V.3	2018	Kenya	MW342782/ck/KE/Kilifi/Sokoni/3FB31/2018/(V.3)	
		MW342788	V.3	2018	Kenya	MW342788/ck/KE/Kilifi/Rabai/3FB128/2018/(V.3)	
		MW342789	V.3	2017	Kenya	MW342789/ck/KE/Nairobi/Burma/A148/2017/(V.3)	
		MW342790	V.3	2017	Kenya	MW342790/ck/KE/Bomet/Kapkwen/34MB09/2017/(V.3)	
		MW342791	V.3	2017	Kenya	MW342791/ck/KE/Nairobi/Burma/A144/2017/(V.3)	
		MW342792	V.3	2018	Kenya	MW342792/ck/KE/Mombasa/Changamwe/1MB35/2018/(V.3)	
		MW342793	V.3	2018	Kenya	MW342793/ck/KE/Mombasa/Changamwe/1MB40/2018/(V.3)	
		MW342794	V.3	2018	Kenya	MW342794/ck/KE/Mombasa/Changamwe/1MB23/2018/(V.3)	
		MW342795	V.3	2018	Kenya	MW342795/ck/KE/Mombasa/Changamwe/1MB42/2018/(V.3)	
		MW342796	V.3	2017	Kenya	MW342796/ck/KE/Nairobi/Kariobangi_North/A369/2017/(V.3)	
		MW342797	V.3	2018	Kenya	MW342797/ck/KE/Kilifi/3FB16/2018/(V.3)	
		MW342798	V.3	2018	Kenya	MW342798/ck/KE/Kilifi/3FB15/2018/(V.3)	
		MW342799	V.3	2018	Kenya	MW342799/ck/KE/Kilifi/MwembeKuku/3MB14/2018/(V.3)	
		MW342800	V.3	2017	Kenya	MW342800/ck/KE/Nairobi/Kariobangi_North/A375/2017/(V.3)	

		MW342801	V.3	2018	Kenya	MW342801/ck/KE/Kilifi/Mtwapa/3FB129/2018/(V.3)	
		MW342802	V.3	2018	Kenya	MW342802/ck/KE/Kilifi/MwembeKuku/3MB09/2018/(V.3)	
		MW342803	V.3	2017	Kenya	MW342803/ck/KE/Nairobi/Burma/A101/2017/(V.3)	
		MW342804	V.3	2017	Kenya	MW342804/ck/KE/Nairobi/Burma/A105/2017/(V.3)	
		MW342805	V.3	2017	Kenya	MW342805/ck/KE/Nairobi/Burma/A104/2017/(V.3)	
		MW342806	V.3	2017	Kenya	MW342806/ck/KE/Nairobi/Burma/A142/2017/(V.3)	
		MW342807	V.3	2017	Kenya	MW342807/ck/KE/Nairobi/Kangemi/A5136/2017/(V.3)	
	**4**	AY288997	V	1994	Kenya	Chicken/Kenya/KRC-150/94	(Pedersen *et al.*, 2004)
**Sudan**	**1**	GQ258675	VII.1.1	2004	Sudan	Chicken/Sudan/08/2004	(Hassan *et al.*, 2010)
		GQ258672	VII.1.1	2004	Sudan	Chicken/Sudan/05/2004	
		GQ258671	VII.1.1	2003	Sudan	Chicken/Sudan/04/2003	
		GQ258670	VII.1.1	2003	Sudan	Chicken/Sudan/03/2003	
		GQ258673	VII.1.1	2006	Sudan	Chicken/Sudan/06/2006	
		GQ258669	VII.1.1	2005	Sudan	Chicken/Sudan/02/2005	
		GQ258674	IV	1987	Sudan	Chicken/Sudan/Obied/1987	
	**2**	AY151384	VI	1975	Sudan	SD-2/75	(Ujvári *et al.*, 2003)
		AY151383	VI	1975	Sudan	SD-3/75	
		AY151385	VI	1975	Sudan	SD-4/75	
**Uganda**	**1**	LT549451	II	2011	Uganda	NDV/Waterfowl/Uganda/MU138/2011	(Omony *et al.*, 2021)
		LT549452	II	2011	Uganda	NDV/Waterfowl/Uganda/MU150/2011	
		LT549453	II	2011	Uganda	NDV/Waterfowl/Uganda/MU186/2011	

		HG937567	V	2011	Uganda	NDV/Chicken/Uganda/MU001/2011	
		HG937568	V	2011	Uganda	NDV/Chicken/Uganda/MU007/2011	
		HG937569	V	2011	Uganda	NDV/Chicken/Uganda/MU009/2011	
		HG937570	V	2011	Uganda	NDV/Chicken/Uganda/MU010/2011	
		HG937571	V	2011	Uganda	NDV/Chicken/Uganda/MU0138/2011	
		HG937572	V	2011	Uganda	NDV/Chicken/Uganda/MU019/2011	
		HG937573	V	2011	Uganda	NDV/Chicken/Uganda/MU024/2011	
		HG937574	V	2011	Uganda	NDV/Chicken/Uganda/MU026/2011	
		HG937575	V	2011	Uganda	NDV/Chicken/Uganda/MU032/2011	
		HG937576	V	2011	Uganda	NDV/Chicken/Uganda/MU033/2011	
		HG937577	V	2011	Uganda	NDV/Chicken/Uganda/MU035/2011	
		HG937578	V	2011	Uganda	NDV/Chicken/Uganda/MU037/2011	
		HG937579	V	2011	Uganda	NDV/Chicken/Uganda/MU039/2011	
		HG937580	V	2011	Uganda	NDV/Chicken/Uganda/MU040/2011	
		HG937581	V	2011	Uganda	NDV/Chicken/Uganda/MU044/2011	
		HG937582	V	2011	Uganda	NDV/Chicken/Uganda/MU050/2011	
		HG937583	V	2011	Uganda	NDV/Chicken/Uganda/MU056/2011	
		HG937584	V	2011	Uganda	NDV/Chicken/Uganda/MU062/2011	
		HG937585	V	2011	Uganda	NDV/Chicken/UgandaMU069/2011	
		HG937586	V	2011	Uganda	NDV/Chicken/Uganda/MU071/2011	
		HG937587	V	2011	Uganda	NDV/Chicken/Uganda/MU074/2011	
		HG937588	V	2011	Uganda	NDV/Chicken/Uganda/MU084/2011	
		HG937589	V	2011	Uganda	NDV/Chicken/Uganda/MU090/2011	
		HG937590	V	2011	Uganda	NDV/Chicken/Uganda/MU111/2011	
		HG937591	V	2011	Uganda	NDV/Chicken/Uganda/MU113/2011	
	**2**	LT549451	II	2011	Uganda	APMV-1/waterfowl/Uganda/MU138/2011	(Wanyana *et al.*, 2018)
		LT549452	II	2011	Uganda	APMV-1/waterfowl/Uganda/MU150/2011	
		LT549453.	II	2011	Uganda	APMV-1/waterfowl/Uganda/MU186/2011	
	**3**	HG937535	V	2011	Uganda	NDV/chicken/Uganda/MU001/2011	(Byarugaba *et al.*, 2014)

		HG937536	V	2011	Uganda	NDV/chicken/Uganda/MU007/2011	
		HG937537	V	2011	Uganda	NDV/chicken/Uganda/MU009/2011	
		HG937538	V	2011	Uganda	NDV/chicken/Uganda/MU010/2011	
		HG937539	V	2011	Uganda	NDV/chicken/Uganda/MU013/2011	
		HG937540	V	2011	Uganda	NDV/chicken/Uganda/MU014/2011	
		HG937541	V	2011	Uganda	NDV/chicken/Uganda/MU019/2011	
		HG937542	V	2011	Uganda	NDV/chicken/Uganda/MU022/2011	
		HG937573	V	2011	Uganda	NDV/chicken/Uganda/MU024/2011	
		HG937543	V	2011	Uganda	NDV/chicken/Uganda/MU026/2011	
		HG937544	V	2011	Uganda	NDV/chicken/Uganda/MU029/2011	
		HG937545	V	2011	Uganda	NDV/chicken/Uganda/MU030/2011	
		HG937546	V	2011	Uganda	NDV/chicken/Uganda/MU031/2011	
		HG937575	V	2011	Uganda	NDV/chicken/Uganda/MU032/2011	
		HG937576	V	2011	Uganda	NDV/chicken/Uganda/MU033/2011	
		HG937547	V	2011	Uganda	NDV/chicken/Uganda/MU035/2011	
		HG937578	V	2011	Uganda	NDV/chicken/Uganda/MU037/2011	
		HG937548	V	2011	Uganda	NDV/chicken/Uganda/MU039/2011	
		HG937580	V	2011	Uganda	NDV/chicken/Uganda/MU040/2011	
		HG937581	V	2011	Uganda	NDV/chicken/Uganda/MU044/2011	
		HG937582	V	2011	Uganda	NDV/chicken/Uganda/MU050/2011	
		HG937550	V	2011	Uganda	NDV/chicken/Uganda/MU052/2011	
		HG937551	V	2011	Uganda	NDV/chicken/Uganda/MU054/2011	
		HG937552	V	2011	Uganda	NDV/chicken/Uganda/MU056/2011	
		HG937535	V	2011	Uganda	NDV/chicken/Uganda/MU058/2011	
		HG937553	V	2011	Uganda	NDV/chicken/Uganda/MU059/2011	
		HG937584	V	2011	Uganda	NDV/duck/Uganda/MU062/2011	
		HG937554	V	2011	Uganda	NDV/chicken/Uganda/MU069/2011	
		HG937555	V	2011	Uganda	NDV/chicken/Uganda/MU071/2011	
		HG937556	V	2011	Uganda	NDV/chicken/Uganda/MU072/2011	
		HG937557	V	2011	Uganda	NDV/chicken/Uganda/MU074/2011	
		HG937558	V	2011	Uganda	NDV/chicken/Uganda/MU077/2011	

		HG937559	V	2011	Uganda	NDV/chicken/Uganda/MU083/2011	
		HG937560	V	2011	Uganda	NDV/chicken/Uganda/MU084/2011	
		HG937561	V	2011	Uganda	NDV/chicken/Uganda/MU090/2011	
		HG937562	V	2011	Uganda	NDV/chicken/Uganda/MU091/2011	
		HG937563	V	2011	Uganda	NDV/chicken/Uganda/MU096/2011	
		HG937564	V	2011	Uganda	NDV/chicken/Uganda/MU098/2011	
		HG937565	V	2011	Uganda	NDV/chicken/Uganda/MU108/2011	
		HG937566	V	2011	Uganda	NDV/chicken/Uganda/MU111/2011	
		HG937591	V	2011	Uganda	NDV/chicken/Uganda/MU113/2011	
	**4**	AY367559	VI.2.1.1.1	2001	Uganda	Chicken/Pallisa/0405/01	(Otim *et al.*, 2004)
		AY371991	VI.2.1.1.1	2001	Uganda	Chicken/Pallisa/0208/01	
		AY371992	VI.2.1.1.1	2001	Uganda	Chicken/Soroti/0104/01	
		AY371993	VI.2.1.1.1	2001	Uganda	Chicken/Pallisa/0406/01	
		AY371994	VI.2.1.1.1	2001	Uganda	Chicken/Pallisa/0321/01	
		AY371995	VI.2.1.1.1	2001	Uganda	Chicken/Pallisa/0305/01	
		AY371996	VI.2.1.1.1	2001	Uganda	Chicken/Soroti/0509/01	
		AY371998	VI.2.1.1.1	2001	Uganda	Chicken/Tororo/0222/01	
		AY371999	VI.2.1.1.1	2001	Uganda	Chicken/Pallisa/0405/01	
		AY372000	VI.2.1.1.1	2001	Uganda	Chicken/Pallisa/0408/01	
		AY372001	VI.2.1.1.1	2001	Uganda	Chicken/Soroti/0814/01	
		AY372002	VI.2.1.1.1	2001	Uganda	Chicken/Pallisa/0609/01	

		AY372003	VI.2.1.1.1	2001	Uganda	Chicken/Pallisa/0206/01	
		AY372004	VI.2.1.1.1	2001	Uganda	Chicken/Pallisa/0102/01.	
		AY372005	VI.2.1.1.1	2001	Uganda	Chicken/Pallisa/0407/01	
		AY372006	VI.2.1.1.1	2001	Uganda	Chicken/Pallisa/0515/01	
		AY371997	VI.2.1.1.1	2001	Uganda	Chicken/Pallisa/0103/01	
**Burundi**	**1**	FJ772490	XIII.1.1	2008	Burundi	chicken-4132-12-Burundi-2008	(Cattoli *et al.*, 2010)
		FJ772493	XIII.1.1	2008	Burundi	chicken-4132-20-Burundi-2008	
**Ethiopia**	**1**		VI	1976	Ethiopia	APMV1/Chk/ETH/R1825/09/1976	(Bari *et al.*, 2021)
			VI	1984	Ethiopia	APMV1/Chk/ETH/R655/08/1984	
			VI	1997	Ethiopia	APMV1/Chk/ETH/R645/08/1997	
			VI	1997	Ethiopia	APMV1/Chk/ETH/R647/08/1997	
			VI	1997	Ethiopia	APMV1/Chk/ETH/R652/08/1997	
			VI	1997	Ethiopia	APMV1/Chk/ETH/R656/08/1997	
			VI	1997	Ethiopia	APMV1/Chk/ETH/R657/08/1997	
			VI	2000	Ethiopia	APMV1/Chk/ETH/R653/08/2000	
			VI	2002	Ethiopia	APMV1/Chk/ETH/R644/08/2002	
			VI	2007	Ethiopia	APMV1/Chk/ETH/R650/08/2007	
	**2**	KR014202	VI.2.1.1.1	2014	Ethiopia	APMV-1/Pigeon/Ethiopia/14VIR4296-2/2773/14/2/2014	(Damena *et al.*, 2016)
		KR014203	VI.2.1.1.1	2014	Ethiopia	APMV-1/Pigeon/Ethiopia/14VIR4296-4/2773/14/4/2014	
		KR014204	VI.2.1.1.1	2014	Ethiopia	APMV-1/Pigeon/Ethiopia/14VIR4296-6/2773/14/6/2014	
		KR014205	VI.2.1.1.1	2014	Ethiopia	APMV-1/Pigeon/Ethiopia/14VIR4296-10/2773/14/10/2014	
		KR014206	VI.1.2.2.1	2012	Ethiopia	APMV-1/Chicken/Ethiopia/14VIR4296-20/1294/2012	

		KR014207	VI.1.2.2.1	2012	Ethiopia	APMV-1/Chicken/Ethiopia/14VIR4296-21/2576/2012	
		KR014208	VI.1.2.2.1	2014	Ethiopia	APMV-1/Chicken/Ethiopia/14VIR4296-22/3083/2014	
		KR014209	VI.1.2.2.1	2014	Ethiopia	APMV-1/Chicken/Ethiopia/14VIR4296-23/15925/2014	
		KR014210	VI.1.2.2.1	2013	Ethiopia	APMV-1/Chicken/Ethiopia/14VIR4296-24/19768/2013	
		KR014211	VI.1.2.2.1	2013	Ethiopia	APMV-1/Chicken/Ethiopia/14VIR4296-25/20045/2013	
		KR014212	VI.1.2.2.1	2013	Ethiopia	APMV-1/Chicken/Ethiopia/14VIR4296-26/20112/2013	
		KR014213	VI.1.2.2.1	2013	Ethiopia	APMV-1/Chicken/Ethiopia/14VIR4296-27/35828/2013	
	**3**	KC851841	VII		Ethiopia	12RS1402-39/APMV1/CK/ Ethiopia	(Fentie *et al.*, 2014)
		KC851842	II		Ethiopia		
