# Supplementary material for: Genomic Diversity and Geographic Distribution of Newcastle Disease Virus Genotypes in Africa: Implications for Diagnosis, Vaccination, and Regional Collaboration
Source: Viruses. 2024 May 16;16(5):795. doi: 10.3390/v16050795 (PMC11125703; doi:10.3390/v16050795)
Supplement: Supplementary file 1 [file viruses-16-00795-s001.zip › Table S2 Distribution of class II NDV genotypes in Southern Africa.pdf]

Table S2: Distribution of class II NDV genotypes in Southern Africa

Country	Paper	Isolate GenBank accession number	Genotype	Year of collection	Country	Isolate name	Reference
**South Africa**	**1**	AY210492	II	1995	South Africa, district unknown	ZA1454/UP/95	(Abolnik *et al.*, 2004)
		AY210489	II	1997	Arnot	ZA719/UP/97	
		AF532145	II	1996	KwaZulu/Natal	ZA202/B/96	
		AF532149	II	1999	Dundee	ZA307/B/99	
		AF532152	II	1999	Port Shepstone area	ZA312/B/99	
		AY210490	II	1999	Hartebeespoort Dam area	ZA1366/UP/99	
		AF532741	I	1999	Port Shepstone area	ZA340/P/99	
		AY210491	II	2000	Pretoria	ZA680/UP/00	
		AF532744	II	2000	Camperdown area	ZA375/B/00	
		AF532742	I	2000	Estcourt	ZA370/B/00	
		AY210493	II	2001	Worchester	ZA12150/Worc/01	
		AF532747	II	2001	Estcourt	ZA393/B/01	
		AF532749	II	2001	Camperdown area	ZA405/BB/01	
		AF532746	II	2001	Camperdown area	ZA385/B/01	
		AF532743	I	2001	Gauteng	ZA37401/X/01	
		AF532142	VIII	1991	Richmond	ZA13/L/91	
		AF136766	VIII	1990	South Africa, district unknown	ZA-18/90	
		AF136775	VIII	1990	South Africa, district unknown	ZA-17/90	
		AF532143	VIII	1991	Richmond	ZA16/GF/91	
		AF532752	VIII	1991	Lion’s River/Merrivale	ZA19/B/91	
		AF532140	VIII	1991	Pietermaritzburg/Thornville	ZA11/B/91	
		AF136773	VIII	1994	South Africa, district unknown	ZA-34/94	
		AF352139	VII.1.1	1994	Estcourt	ZA108/L/94	
		AF532751	VII.1.1	1994	Camperdown area	ZA71/B/94	
		AF532750	VII.1.1	1994	Camperdown area	ZA60/B/94	

		AF532141	VII.1.1	1994	Pretoria	ZA110/X/94	
		AF532749	VII.1.1	1994	Camperdown area	ZA52/BB/94	
		AF532144	VII.1.1	1995	Lower Tugela/Umhlali	ZA170/B/95	
		AF136774	VII.1.1	1995	South Africa, district unknown	ZA-35/95	
		AF109884	VII.1.1	1996	United Arab Emirates	AE232/1/96	
		AY210494	VII.1.1	1997	Kuruman	ZA839/UP/97	
		AY210504	VII.1.1	1997	Pretoria	ZA842/UP/97	
		AY210499	VII.1.1	1997	Potgietersrus	ZA825/UP/97	
		AF532146	VII.1.1	1997	Pretoria	ZA256/X/97	
		AY210505	VII.1.1	1998	Pretoria	ZA751/UP/98	
		AY210506	VII.1.1	1998	Rustenburg	ZA874/UP/98	
		AY210508	VII.1.1	1998	Rustenburg	ZA704/UP/98	
		AY210509	VII.1.1	1998	Rustenburg	ZA983/UP/98	
		AY210511	VII.1.1	1998	Hammarsdale	ZA444/B/98	
		AY210502	VII.1.1	1998	Riversdale	ZA7351/Rvsdl/98	
		AF532148	VII.1.1	1998	Durban	ZA296/L/98	
		AY210512	VII.1.1	1998	Western Cape	ZA7381/98	
		AF532739	VII.1.1	1999	Stanger	ZA331/B/99	
		AF532150	VII.1.1	1999	New Hanover/Wartburg	ZA308/B/99	
		AY210510	VII.1.1	1999	Pretoria	ZA1320/UP/99	
		AY210501	VII.1.1	1999	Halfway House	ZA1521/UP/99	
		AF532740	VII.1.1	1999	Camperdown area	ZA335/B/99	
		AY210500	VII.1.1	1999	Potchefstroom	ZA549/UP/99	
		AF532151	VII.1.1	1999	Pietersburg	ZA309/B/99	
		AY210503	VII.1.1	1999	Klapmuts	ZA3291/Klpmts/99	
		AY210496	VIII	2000	Pretoria	ZA598/UP/00	
		AY210497	VIII	2000	Pretoria	ZA606/UP/00	
		AF532745	VII.1.1	2000	Port Shepstone area	ZA378/F/00	
	**2**	MF622035	VII.2	Aug-13	South Africa	Chicken/South Africa/230665/2013	(Abolnik *et al.*, 2018)
		KR815908	VII.2	Oct-13	South Africa	Turkey/South Africa/N2057/2013	

		MF622045	VII.2	Nov-13	South Africa	Chicken/South Africa/RBNW-1/2013	
		MF622046	VII.2	Nov-13	South Africa	Chicken/South Africa/RBNW-2/2013	
		MF622047	VII.2	Nov-13	South Africa	Chicken/South Africa/RBNW-3/2013	
		MF622040	VII.2	Nov-13	South Africa	Chicken/South Africa/Inchanga/2013	
		MF622037	VII.2	Nov-13	South Africa	Chicken/South Africa/239391/2013	
		MF622039	VII.2	Sep-14	South Africa	Chicken/South Africa/H14973/2014	
		MF622034	VII.2	Jul-15	South Africa	Chicken/South Africa/32995/2015	
		MF622043	VII.2	Aug-15	South Africa	Chicken/South Africa/N2117/2015	
		MF622044	VII.2	Nov-15	South Africa	Chicken/South Africa/N2683/2015	
	**3**	AF136762	VIII	1968	South Africa	ZA-5/68	(Herczeg *et al.*, 1999)
		AF136763	VIII	1974	South Africa	ZA-10/74	
		AF136764	VIII	1990	South Africa	ZA-16/90	
		AF136775	VIII	1990	South Africa	ZA-17/90	
		AF136766	VIII	1990	South Africa	ZA-18/90	
		AF136767	VII.1.1	1993	South Africa	ZA-20/93	
		AF136768	VII.1.1	1993	South Africa	ZA-25/93	
		AF136769	VII.1.1	1993	South Africa	ZA-26/93	
		AF136770	VII.1.1	1993	South Africa	ZA-29/93	
		AF136771	VII.1.1	1993	South Africa	ZA-32/93	
		AF136772	VII.1.1	1994	South Africa	ZA-33/94	
		AF136773	VIII	1994	South Africa	ZA-34/94	
		AF136774	VII.1.1	1995	South Africa	ZA-35/95	
**Mozambique**	**1**	KX231366	VII.2	2011	Mozambique	NDV/chicken/ Mozambique/1205/2011	(Mapaco *et al.*, 2016)
		KU523524	VII.2	2012	Mozambique	NDV/chicken/ Mozambique/466/2012	
		KU523526	VII.2	2012	Mozambique	NDV/chicken/ Mozambique/491/2012	

		KU523528	VII.2	2012	Mozambique	NDV/chicken/ Mozambique/658/2012	
		KU523529	VII.2	2013	Mozambique	NDV/chicken/ Mozambique/584/2013	
		MF622036	VII.2	2013	Mozambique	Chicken/Zimbabwe/ 235280/2013	
		KX231367	VII.2	2014	Mozambique	NDV/chicken/ Mozambique/475/2014	
		KU523533	VII.2	2014	Mozambique	NDV/chicken/ Mozambique/622/2014	
		KX231368	VII.2	2016	Mozambique	NDV/chicken/ Mozambique/192A/2016	
	**2**	HQ702462	I	2005	Mozambique	chicken/Mozambique/ndv42_564/ 2005	(Snoeck *et al.*, 2009)
	**3**	AF136775	VII.1.1	1994	Mozambique	MZ 13/94	(Herczeg *et al.*, 1999)
		AF136776	VII.1.1	1995	Mozambique	MZ 35/94	
		AF136777	VII.1.1	1995	Mozambique	MZ-44/95	
		AF136778	VII.1.1	1995	Mozambique	MZ-46/95	
		AF136779	VII.1.1	1995	Mozambique	MZ-48/95	
**Botswana**	**1**	MT091950	VII.2	2014	Botswana	NDV/Chicken/Botswana/1/2014	(Kgotlele *et al.*, 2020)
		MT091951	VII.2	2014	Botswana	NDV/Chicken/Botswana/2/2014	
		MT091952	VII.2	2014	Botswana	NDV/Chicken/Botswana/4/2014	
		MT091953	VII.2	2018	Botswana	NDV/Chicken/Botswana/6/2018	
		MT091954	VII.2	2018	Botswana	NDV/Chicken/Botswana/7/2018	
		MT091955	VII.2	2018	Botswana	NDV/Chicken/Botswana/8/2018	
		MT091956	VII.2	2018	Botswana	NDV/Chicken/Botswana/9/2018	
		MT091957	VII.2	2018	Botswana	NDV/Chicken/Botswana/10/2018	
		MT091958	VII.2	2019	Botswana	NDV/Chicken/Botswana/11/2019	
		MT091959	VII.2	2019	Botswana	NDV/Chicken/Botswana/12/2019	
		MT091960	VII.2	2019	Botswana	NDV/Chicken/Botswana/14/2019	
		MT091961	VII.2	2019	Botswana	NDV/Chicken/Botswana/15/2019	
		MT091962	VII.2	2019	Botswana	NDV/Chicken/Botswana/16/2019	
		MT091963	VII.2	2019	Botswana	NDV/Chicken/Botswana/18/2019	

**Namibia**	**1**	KY747479	VII.2	2016	Namibia	NDV/Chicken/Namibia/5620/2016	(Molini *et al*., 2017)
		KY747480	VII.2	2016	Namibia	NDV/Chicken/Namibia/6195/2016	
		KY747481	VII.2	2016	Namibia	NDV/Chicken/Namibia/6196/2016	
		KY747482	VII.2	2016	Namibia	NDV/Chicken/Namibia/6403/2016	
		KY747483	VII.2	2016	Namibia	NDV/Chicken/Namibia/6762/2016	
		KY747484	VII.2	2016	Namibia	NDV/Chicken/Namibia/7362/2016	
**Zambia**	**1**	MF409241	XIII	2015	Zambia	chicken/Zambia/Chiwoko/2015	(Abolnik *et al*., 2017)
	**2**	MF622041	VII.2	2015	Zambia	Chicken/Zambia/Katete/ 2015	(Abolnik *et al*., 2018)
		MF622042	VII.2	2015	Zambia	Chicken/Zambia/ Mbeweka/2015	
		MF622038	VII.2	2015	Zambia	Chicken/Zambia/Chadiza/ 2015	
**Zimbabwe**	**1**	AY175710	II	1995	Zimbabwe	AV 862/95 5249	(Aldous *et al*., 2003)
	**2**	MF622036	VII.2	2013	Zimbabwe	chicken/Zimbabwe/235280/2013	(Abolnik *et al*., 2018)

References

Abolnik, C., Horner, R. F., Bisschop, S. P. R., Parker, M. E., Romito, M., & Viljoen, G. J. (2004). A phylogenetic study of South African Newcastle disease virus strains isolated between 1990 and 2002 suggests epidemiological origins in the Far East. *Archives of Virology*, *149*(3), 603–619. https://doi.org/10.1007/s00705-003-0218-2

Abolnik, C., Mubamba, C., Wandrag, D. B. R., Horner, R., Gummow, B., Dautu, G., & Bisschop, S. P. R. (2018). Tracing the origins of genotype VIIh Newcastle disease in southern Africa. *Transboundary and Emerging Diseases*, *65*(2), e393–e403. https://doi.org/10.1111/tbed.12771

Abolnik, Celia, Mubamb, C., Dautu, G., & Gummow, B. (2017). Complete genome sequence of a Newcastle disease genotype XIII virus isolated from indigenous chickens in Zambia. *Genome Announcements*, *5*(34). https://doi.org/10.1128/genomeA.00841-17

Aldous, E. W., Mynn, J. K., Banks, J., & Alexander, D. J. (2003). A molecular epidemiological study of avian paramyxovirus type 1 (Newcastle disease virus) isolates by phylogenetic analysis of a partial nucleotide sequence of the fusion protein gene. *Avian Pathology*, *32*(3), 239–257. https://doi.org/10.1080/030794503100009783

Herczeg, J., Wehmann, E., Bragg, R. R., Travassos Dias, P. M., Hadjiev, G., Werner, O., & Lomniczi, B. (1999). Two novel genetic groups (VIIb and VIII) responsible for recent Newcastle disease outbreaks in Southern Africa, one (VIIb) of which reached Southern Europe. *Archives of Virology*, *144*(11), 2087–2099. https://doi.org/10.1007/s007050050624

Kgotlele, T., Modise, B., Nyange, J. F., Thanda, C., Cattoli, G., & Dundon, W. G. (2020). First molecular characterization of avian paramyxovirus-1 (Newcastle disease virus) in Botswana. *Virus Genes*, *56*(5), 646–650. https://doi.org/10.1007/s11262-020-01770-4

Mapaco, L. P., Monjane, I. V. A., Nhamusso, A. E., Viljoen, G. J., Dundon, W. G., & Achá, S. J. (2016). Phylogenetic analysis of Newcastle disease viruses isolated from commercial poultry in Mozambique (2011–2016). *Virus Genes*, *52*(5), 748–753. https://doi.org/10.1007/s11262-016-1362-6

Molini, U., Aikukutu, G., Khaiseb, S., Cattoli, G., & Dundon, W. G. (2017). First genetic characterization of newcastle disease viruses from Namibia: identification of a novel VIIk subgenotype. *Archives of Virology*, *162*(8), 2427–2431. https://doi.org/10.1007/s00705-017-3389-y

Snoeck, C. J., Ducatez, M. F., Owoade, A. A., Faleke, O. O., Alkali, B. R., Tahita, M. C., Tarnagda, Z., Ouedraogo, J. B., Maikano, I., Mbah, P. O., Kremer, J. R., & Muller, C. P. (2009). Newcastle disease virus in West Africa: New virulent strains identified in non-commercial farms. *Archives of Virology*, *154*(1), 47–54. https://doi.org/10.1007/s00705-008-0269-5
