# Supplementary material for: Genomic Diversity and Geographic Distribution of Newcastle Disease Virus Genotypes in Africa: Implications for Diagnosis, Vaccination, and Regional Collaboration
Source: Viruses. 2024 May 16;16(5):795. doi: 10.3390/v16050795 (PMC11125703; doi:10.3390/v16050795)
Supplement: Supplementary file 1 [file viruses-16-00795-s001.zip › Table S3 Distribution of class II NDV genotypes in Western Africa.pdf]

Table S3: Distribution of class II NDV genotypes in Western Africa

Country	Paper	Isolate GenBank accession number	Genotype	Year of collection	Country	Isolate name	Reference
**Côte d'Ivoire**	**1**	FJ772466	XVII	2008	Ivory Coast	chicken/Ivory Coast/2601/2008	(Cattoli *et al.*, 2010)
	**2**	HF969127	XVIII.2	2007	Ivory Coast	chicken/Ivory Coast/CIV08-069/2007	(Snoeck, *et al.*, 2013)
		HF969126	XVIII.2	2006	Ivory Coast	duck/Ivory Coast/CIV08-062/2006	
		HF969179	XVIII.1	2007	Ivory Coast	chicken Ivory Coast/CIV08-026/2007	
		HF969218	XVIII.2	2007	Ivory Coast	chicken/Ivory Coast/CIV08-042/2007	
		HF969183	XVIII.1	2007	Ivory Coast	chicken/Ivory Coast/CIV08-044/2007	
		HF969185	XVII	2007	Ivory Coast	chicken/Ivory Coast/CIV08-104/2007	
	**3**	HG326600	XVIII	2006	Ivory Coast	village weaver/Ivory Coast/CIV08-032/2006	(Snoeck, *et al.*, 2013)
**Benin**	**1**	JX546255	VII	2009	Benin	APMV-1/chicken/Benin/432MT/2009	(Samuel *et al.*, 2013)
		JX546256	VII	2009	Benin	APMV-1/chicken/Benin/467MC/2009	
		JX546257	VII	2009	Benin	APMV-1/chicken/Benin/476MT/2009	
		JX546258	VII	2009	Benin	APMV-1/chicken/Benin/480MT/2009	
		JX546259	VII	2009	Benin	APMV-1/chicken/Benin/415MC/2009	
		JX546246	VII	2009	Benin	APMV-1/chicken/Benin/474MC/2009	
		JX546260	VII	2009	Benin	APMV-1/chicken/Benin/442MT/2009	
		JX546261	VII	2009	Benin	APMV-1/chicken/Benin/479MT/2009	
		JX546262	VII	2009	Benin	APMV-1/chicken/Benin/382GT/2009	
		JX546263	VII	2009	Benin	APMV-1/chicken/Benin/847GC/2009	
		JX546264	VII	2009	Benin	APMV-1/chicken/Benin/380GC/2009	
		JX546265	VII	2009	Benin	APMV-1/chicken/Benin/372GC/2009	
		JX546266	VII	2009	Benin	APMV-1/chicken/Benin/796GT/2009	
		JX546267	VII	2009	Benin	APMV-1/chicken/Benin/349GC/2009	
		JX546268	VII	2009	Benin	APMV-1/chicken/Benin/770GT/2009	
		JX546269	VII	2009	Benin	APMV-1/chicken/Benin/379GT/2009	
		JX546270	VII	2009	Benin	APMV-1/chicken/Benin/378GT/2009	

		JX546271	VII	2009	Benin	APMV-1/chicken/Benin/846GC/2009	
		JX546245	VII	2009	Benin	APMV-1/chicken/Benin/463MT/2009	
		JX546247	VII	2009	Benin	APMV-1/chicken/Benin/488MT/2009	
		JX546244	VII	2009	Benin	APMV-1/chicken/Benin/376GT/2009	
		JX546243	VII	2009	Benin	APMV-1/chicken/Benin/373GC/2009	
	**2**	JX546245	XIV.2	2009	Benin	chicken/Benin/463MT/2009	(Snoeck *et al.*, 2013)
		JX546243	XVII	2009	Benin	chicken/Benin/373GC/2009	
		JX546244	XVII	2009	Benin	chicken/Benin/376GT/2009	
		JX546247	XVII	2009	Benin	chicken/Benin/488MT/2009	
**Burkina Faso**	**1**	FM200808	XIV	2006	Burkina Faso	chicken/Burkina Faso/SKF4.1/2006	(Snoeck *et al.*, 2009)
		FM200806	XIV	2006	Burkina Faso	chicken/Burkina Faso/SKF4.2/2006	
		FM200832	XIV	2006	Burkina Faso	duck/Burkina Faso/C3/2006	
		FM200807	XIV	2006	Burkina Faso	chicken/Burkina Faso/SKF1.2/2006	
		FM200827	XVII	2006	Burkina Faso	chicken/Burkina Faso/5.11/2006	
		FM200805	XVII	2006	Burkina Faso	chicken/Burkina Faso/5.18/2006	
		FM200804	XVII	2006	Burkina Faso	chicken/Burkina Faso/5.2/2006	
		FM200830	II	2006	Burkina Faso	chicken/Burkina Faso/9.12/2006	
		FM200828	II	2006	Burkina Faso	chicken/Burkina Faso/9.2/2006	
		FM200829	II	2006	Burkina Faso	chicken/Burkina Faso/9.8/2006	
		FM200826	II	2006	Burkina Faso	chicken/Burkina Faso/5.5/2006	
	**2**	FJ772463	XVIII.1	2008	Burkina Faso	Burkina Faso/2415-580/2008	(Cattoli *et al.*, 2010)
		FJ772458	XVIII.1	2008	Burkina Faso	Burkina Faso/2415-361/2008	
**Ghana**	**1**	MT335751	XVIII.2	2018	Ghana	NE-29A-B2/chicken/Pokoasi/Ghana/2018	(da Silva *et al.*, 2020)
		MT335752	XVIII.2	2018	Ghana	NE-28A-B1/chicken/Wa/Ghana/2018	
		MT335753	XVIII.2	2018	Ghana	NE-28A-B2/chicken/Wa/Ghana/2018	
		MT335754	XVIII.2	2018	Ghana	NE-28A-B3/chicken/Wa/Ghana/2018	
**Mali**	**1**	JF966385	XIV.1	2008	Mali	2008/Mali/ML007/08	(de Almeida *et al.*, 2013)
		JF966386	XIV	2007	Mali	2007/Mali/ML029/07	
		JF966387	XIV.2	2009	Mali	2009/Mali/ML008	
		JF966388	XIV.2	2008	Mali	2008/Mali/ML225/08	
		JF966389	XIV.2	2007	Mali	2007/Mali/ML038/07	

		JX518885	XIV.2	2010	Mali	2010/Mali/ML57051T	
		JX518886	XIV.2	2010	Mali	2010/Mali/ML57072T	
**Mauritania**	**1**	FJ772455	XVII	2006	Mauritania	avian-1532-14-Mauritania-2006	(Cattoli *et al.*, 2010)
**Niger**	**1**	FM200838	XIV.1	2007	Niger	chicken/Niger/47/2007	(Snoeck *et al.*, 2009)
		FM200836	XIV.1	2007	Niger	chicken/Niger/36/2007	
		FM200809	XIV.1	2007	Niger	chicken/Niger/38/2007	
		FM200837	XIV.1	2007	Niger	chicken/Niger/46/2007	
		FM200835	XVII	2006	Niger	duck/Niger/30/2007	
		FM200833	XVII	2006	Niger	duck/Niger/28/2007	
		FM200834	XVII	2006	Niger	duck/Niger/29/2007	
	**2**	FJ772452	XIV	2006	Niger	chicken-1377-8-Niger-2006	(Cattoli *et al.*, 2010)
		FJ772469	XIV	2008	Niger	chicken-2602-348-Niger-2008	
		FJ772472	XIV	2008	Niger	chicken-2602-468-Niger-2008	
		FJ772475	XIV	2008	Niger	chicken-2602-605-Niger-2008	
		FJ772481	XIV	2008	Niger	chicken-2602-625-Niger-2008	
	**3**	MT543153	XIV.2	2019	Niger	NDV/Chicken/Niger/89/2019	(Souley *et al.*, 2021)
		MT543154	XVIII.1	2019	Niger	NDV/Chicken/Niger/221/2019	
		MT543155	XVIII.1	2019	Niger	NDV/Chicken/Niger/222/2019	
		MT543156	XVIII.1	2019	Niger	NDV/Chicken/Niger/224/2019	
		MT543157	XVIII.1	2019	Niger	NDV/Chicken/Niger/248/2019	
		MT543158	XVIII.1	2019	Niger	NDV/Chicken/Niger/250/2019	
		MT543159	XIV.2	2019	Niger	NDV/Chicken/Niger/255/2019	
		MT543160	XIV.2	2019	Niger	NDV/Chicken/Niger/261/2019	
		MT543161	XIV.2	2019	Niger	NDV/Chicken/Niger/262/2019	
		MT543162	XIV.2	2019	Niger	NDV/Chicken/Niger/270/2019	
**Nigeria**	**1**	FJ772486	XVIII.1	2008	Nigeria	avian-3724-6-Nigeria-2008	(Cattoli *et al.*, 2010)
		FJ772446	XIV.1	2006	Nigeria	avian-913-1-Nigeria-2006	
		FJ772449	XIV.1	2006	Nigeria	avian-913-33-Nigeria-2006	
	**2**	HG424626	XXI.2	2013	Nigeria	pigeon/Nigeria/NIE13-008/2013	(Snoeck *et al.*, 2013)
		HG424625	XXI.2	2013	Nigeria	pigeon/Nigeria/NIE13-005/2013	
		HG326602	VI.1.2.1.2	2007	Nigeria	pigeon/Nigeria/NIE07-062/2007	
		HG326601	VI.1.2.1.2	2007	Nigeria	pigeon/Nigeria/NIE07-061/2007	
		HG326603	VI.1.2.1.2	2007	Nigeria	pigeon/Nigeria/NIE07-063/2007	
		HG326604	VI.1.2.1.2	2009	Nigeria	pigeon/Nigeria/NIE09-1898/2009	
		HG424628	VI.1.2.1.2	2013	Nigeria	pigeon/Nigeria/NIE13-093/2013	
		HG424627	VI.1.2.1.2	2013	Nigeria	pigeon/Nigeria/NIE13-092/2013	
		HG326606	I	2008	Nigeria	spur-winged goose/Nigeria/NIE08-0124/2008	
		HG326608	I	2008	Nigeria	spur-winged goose/Nigeria/NIE08-0273/2008	

		HG326605	I	2008	Nigeria	spur-winged goose/Nigeria/NIE08-0121/2008	
		HG326607	I	2008	Nigeria	spur-winged goose/Nigeria/NIE08-0232/2008	
		HG424629	VI.1.2.1.2	2013	Nigeria	pigeon/Nigeria/NIE13-009/2013	
		HG424627	VI.1.2.1.2	2013	Nigeria	pigeon/Nigeria/NIE13-092/2013	
		HG424628	VI.1.2.1.2	2013	Nigeria	pigeon/Nigeria/NIE13-093/2013	
		HG326603	VI.1.2.1.2	2007	Nigeria	pigeon/Nigeria/NIE07-063/2007	
		HG326602	VI.1.2.1.2	2007	Nigeria	pigeon/Nigeria/NIE07-062/2007	
		HG326609	I	2008	Nigeria	spur-winged goose/Nigeria/NIE08-0290/2008	
	**3**	HF969193	XIV.1	2009	Nigeria	chicken/Nigeria/NIE08-2150/2009	(Snoeck *et al.*, 2013)
		HF969136	XIV.1	2009	Nigeria	chicken/Nigeria/NIE08-2194/2009	
		HF969131	XIV.1	2009	Nigeria	chicken/Nigeria/NIE08-2117/2009	
		HF969200	XIV.1	2009	Nigeria	chicken/Nigeria/NIE08-2362/2009	
		HF969139	XIV.1	2009	Nigeria	chicken/Nigeria/NIE08-2280/2009	
		HF969201	XIV.2	2009	Nigeria	chicken/Nigeria/NIE09-1599/2009	
		HF969142	XIV.2	2009	Nigeria	chicken/Nigeria/NIE09-1596/2009	
		HF969143	XIV.2	2009	Nigeria	chicken/Nigeria/NIE09-1597/2009	
		HF969155	XIV.1	2009	Nigeria	chicken/Nigeria/NIE09-2087/2009	
		HF969158	XIV.1	2009	Nigeria	avian/Nigeria/NIE09-2168/2009	
		HF969206	XIV.1	2009	Nigeria	chicken/Nigeria/NIE09-2101/2009	
		HF969153	XIV.1	2009	Nigeria	chicken/Nigeria/NIE09-2079/2009	
		HF969150	XIV.1	2009	Nigeria	chicken/Nigeria/NIE09-2044/2009	
		HF969205	XIV.1	2009	Nigeria	turkey/Nigeria/NIE09-2071/2009	
		HF969144	XIV.1	2009	Nigeria	chicken/Nigeria/NIE09-2009/2009	
		HF969167	XIV.1	2011	Nigeria	turkey/Nigeria/NIE10-082/2011	
		HF969190	XIV.2	2009	Nigeria	chicken/Nigeria/NIE08-2032/2009	
		HF969187	XIV.2	2009	Nigeria	chicken/Nigeria/NIE08-0453/2008	
		HF969141	XIV.2	2009	Nigeria	chicken/Nigeria/NIE08-2359/2009	
		HF969178	XIV.2	2009	Nigeria	chicken/Nigeria/NIE08-2270/2009	
		HF969133	XIV.2	2009	Nigeria	chicken/Nigeria/NIE08-2159/2009	
		HF969198	XIV.2	2013	Nigeria	chicken/Nigeria/NIE08-2279/2009	
		HF969212	XIV.2	2009	Nigeria	chicken/Nigeria/NIE10-318/2011	

		HF969145	XIV.2	2009	Nigeria	chicken/Nigeria/NIE09-2014/2009	
		HF969203	XIV.2	2009	Nigeria	turkey/Nigeria/NIE09-2021/2009	
		HF969149	XIV.2	2009	Nigeria	chicken/Nigeria/NIE09-2041/2009	
		HF969157	XIV.2	2009	Nigeria	chicken/Nigeria/NIE09-2166/2009	
		HF969151	XIV.2	2009	Nigeria	chicken/Nigeria/NIE09-2053/2009	
		HF969202	XIV.2	2009	Nigeria	chicken/Nigeria/NIE09-2013/2009	
		HF969146	XIV.2	2009	Nigeria	chicken/Nigeria/NIE09-2017/2009	
		HF969214	XIV.2	2011	Nigeria	chicken/Nigeria/NIE10-333/2011	
		HF969162	XIV.2	2011	Nigeria	chicken/Nigeria/NIE10-032/2011	
		HF969169	XIV.2	2011	Nigeria	chicken/Nigeria/NIE10-150/2011	
		HF969210	XIV.2	2011	Nigeria	chicken/Nigeria/NIE10-139/2011	
		HF969163	XIV.2	2011	Nigeria	chicken/Nigeria/NIE10-034/2011	
		HF969172	XIV.2	2011	Nigeria	chicken/Nigeria/NIE10-258/2011	
		HF969213	XIV.2	2011	Nigeria	chicken/Nigeria/NIE10-325/2011	
		HF969208	XIV.2	2011	Nigeria	chicken/Nigeria/NIE10-122/2011	
		HF969166	XIV.2	2011	Nigeria	chicken/Nigeria/NIE10-076/2011	
		HF969161	XIV.2	2011	Nigeria	chicken/Nigeria/NIE10-024/2011	
		HF969236	XIV.2	2011	Nigeria	chicken/Nigeria/NIE10-409/2011	
		HF969170	XIV.2	2011	Nigeria	chicken/Nigeria/NIE10-160/2011	
		HF969173	XIV.2	2011	Nigeria	chicken/Nigeria/NIE10-302/2011	
		HF969164	XIV.2	2011	Nigeria	chicken/Nigeria/NIE10-041/2011	
		HF969211	XIV.2	2011	Nigeria	chicken/Nigeria/NIE10-263/2011	
		HF969165	XIV.2	2011	Nigeria	chicken/Nigeria/NIE10-043/2011	
		HF969217	XVIII.2	2011	Nigeria	chicken/Nigeria/NIE10-171/2011	
		HF969216	XVIII.2	2011	Nigeria	chicken/Nigeria/NIE11-1286/2011	
		HF969194	XVII	2009	Nigeria	chicken/Nigeria/NIE08-2199/2009	
		HF969196	XVII	2009	Nigeria	chicken/Nigeria/NIE08-2261/2009	
		HF969128	XVII	2007	Nigeria	avian/Nigeria/NIE07-216/2007	
		HF969137	XVII	2009	Nigeria	chicken/Nigeria/NIE08-2208/2009	
		HF969130	XVII	2009	Nigeria	guinea fowl/Nigeria/NIE08-2004/2009	
		HF969138	XVII	2009	Nigeria	chicken/Nigeria/NIE08-2224/2009	
		HF969191	XVII	2009	Nigeria	chicken/Nigeria/NIE08-2042/2009	
		HF969192	XVII	2009	Nigeria	chicken/Nigeria/NIE08-2119/2009	
		HF969135	XVII	2009	Nigeria	chicken/Nigeria/NIE08-2187/2009	
		HF969195	XVII	2009	Nigeria	chicken/Nigeria/NIE08-2247/2009	

		HF969134	XVII	2009	Nigeria	chicken/Nigeria/NIE08-2168/2009	
		HF969197	XVII	2009	Nigeria	chicken/Nigeria/NIE08-2267/2009	
		HF969140	XVII	2009	Nigeria	chicken/Nigeria/NIE08-2340/2009	
		HF969132	XVII	2009	Nigeria	chicken/Nigeria/NIE08-2149/2009	
		HF969141	XVII	2009	Nigeria	chicken/Nigeria/NIE08-2349/2009	
		HF969188	XVII	2008	Nigeria	chicken/Nigeria/NIE08-1365/2008	
		HF969189	XVII	2008	Nigeria	chicken/Nigeria/NIE08-1366/2008	
		HF969129	XVII	2008	Nigeria	chicken/Nigeria/NIE08-1363/2008	
		HF969207	XVII	2009	Nigeria	avian/Nigeria/NIE09-2167/2009	
		HF969148	XVII	2009	Nigeria	chicken/Nigeria/NIE09-2034/2009	
		HF969152	XVII	2009	Nigeria	chicken/Nigeria/NIE09-2072/2009	
		HF969154	XVII	2009	Nigeria	chicken/Nigeria/NIE09-2083/2009	
		HF969204	XVII	2009	Nigeria	chicken/Nigeria/NIE09-2028/2009	
		HF969147	XVII	2009	Nigeria	chicken/Nigeria/NIE09-2031/2009	
		HF969156	XVII	2009	Nigeria	chicken/Nigeria/NIE09-2128/2009	
		HF969215	XVII	2011	Nigeria	chicken/Nigeria/NIE10-335/2011	
		HF969174	XVII	2011	Nigeria	chicken/Nigeria/NIE10-304/2011	
		HF969175	XVII	2011	Nigeria	chicken/Nigeria/NIE10-306/2011	
		HF969176	XVII	2011	Nigeria	chicken/Nigeria/NIE10-310/2011	
		HF969209	XVII	2011	Nigeria	chicken/Nigeria/NIE10-123/2011	
		HF969168	XVII	2014	Nigeria	chicken/Nigeria/NIE10-124/2011	
	**4**	JQ039385	XXI.1.1	2007	Nigeria	Dove/Nigeria/VRD07-163/2007	(van Borm *et al.*, 2012)
		JQ039386	XIV	2008	Nigeria	chicken/Nigeria/VRD08-36/2008	
		JQ039387	VI.1.1	2008	Nigeria	pigeon/Nigeria/VRD08-37BRpe(7-9)/2008	
		JQ039388	VI.1.1	2008	Nigeria	pigeon/Nigeria/VRD08-37(10-11-13)/2008	
		JQ039389	VI.1.1	2007	Nigeria	pigeon/Nigeria/VRD07-369/2007	
		JQ039390	XIV	2007	Nigeria	chicken/Nigeria/VRD07-233/2007	
		JQ039391	VI.1.1	2007	Nigeria	pigeon/Nigeria/VRD07-231/2007	
		JQ039392	XVII	2007	Nigeria	avian/Nigeria/VRD07-733/2007	
		JQ039393	XVII	2007	Nigeria	chicken/Nigeria/VRD07-141/2007	
		JQ039394	XVII	2007	Nigeria	chicken/Nigeria/VRD07-410/2007	
		JQ039395	XXI.1.1	2007	Nigeria	Pigeon/Nigeria/VRD07-173/2007	

		JQ039396	XVII	2007	Nigeria	chicken/Nigeria/VRD07-121B/2007	
	**5**	MH996911	I.1.1	2009	Nigeria	BS/350 (N35)	(Welch *et al.*, 2019)
		MH996910	I.1.1	2009	Nigeria	JN/469 (N44)	
		MH996912	II	2010	Nigeria	BKK/497 (N27)	
		MH996917	II	2002/2003	Nigeria	BN08 (N50)	
		MH996915	II	2009	Nigeria	LTS/08 (N25)	
		MH996916	II	2002/2003	Nigeria	PL JZ04 (N49)	
		MH996914	II	2002/2003	Nigeria	PL038 (N47)	
		MH996913	II	2004	Nigeria	VRD17/04 (N2)	
		MH996920	VI.1.2.1.2	2008	Nigeria	VRD08/385 (N23)	
		MH996942	XIV.2	2009	Nigeria	GM/GMM/17- 18T (N14)	
		MH996919	XIV.2	2009	Nigeria	JG/DT/30-31T (N18)	
		MH996938	XIV.2	2009	Nigeria	JG/SH/47C (N15)	
		MH996930	XIV.2	2009	Nigeria	JN/469 (N44)	
		MH996946	XIV.2	2009	Nigeria	KT/JBY/09T (N40)	
		MH996937	XIV.2	2009	Nigeria	KT/KNK/01T (N13)	
		MH996922	XIV.2	2009	Nigeria	KT/MSH/15C (N2)	
		MH996941	XIV.2	2009	Nigeria	NS/KF/06- 09C (N46)	
		MH996945	XIV.2	2009	Nigeria	VRD09/025 (N21)	
		MH996927	XIV.2	2009	Nigeria	VRD09/340 (N50)	
		MH996923	XIV.2	2009	Nigeria	VRD09/546 (N4)	
		MH996943	XIV.2	2009	Nigeria	YB/GSH1/4- 6T (N3)	
		MH996934	XIV.2	2009	Nigeria	YB/GSHI/07T (N5)	
		MH996921	XVII	2010	Nigeria	AD/WB/12C (N29)	
		MH996918	XVII	2009	Nigeria	BA/BAUR/07T (N17)	
		MH996925	XVII	2009	Nigeria	BA/TFB/14C (N38)	
		MH092811	XVII	2010	Nigeria	BKK/497 (N27)	
		MH996926	XVII	2009	Nigeria	BO/MMC/ AGN/06- 07T (N42)	
		MH996939	XVII	2009	Nigeria	JG/SH/47C (N15)	
		MH092808	XVII	2009	Nigeria	JN/327 (N24)	
		MH996928	XVII	2009	Nigeria	KD/TW/03T (N45)	
		MH996929	XVII	2009	Nigeria	KG/LOM/11- 16 (N11)	
		MH092810	XVII	2009	Nigeria	KN/399 (N26)	
		MH996931	XVII	2009	Nigeria	KT/MA/5-6C (N7)	
		MH092809	XVII	2009	Nigeria	LTS/08 (N25)	
		MH996933	XVII	2009	Nigeria	LTS/11T (N38)	
		MH996940	XVII	2009	Nigeria	NS/KR/60- 61C (N16)	
		MH996932	XVII	2002/2003	Nigeria	PL038 (N47)	
		MH996944	XVII	2009	Nigeria	YB/GSH1/4- 6T (N3)	
		MH996935	XVII	2009	Nigeria	YB/GSHI/07T (N5)	
		MH996936	XVII	2009	Nigeria	YB/GSHI/9- 10C (N9)	
		MH996924	XVII	2009	Nigeria	ZM/KN/ GF01bC (N6)	

		MH392227	XVII	2009	Nigeria	OOT/4/1 (N69)	
	**6**	MN046110	XIV.2	2017	Nigeria	PLJS-109/2017	(Abah *et al.*, 2020)
		MN046107	XIV.2	2009	Nigeria	PLJN-T11/2009	
		MNO46108	XVII	2009	Nigeria	NSKR-DK56-59/2009	
		MN046106	XVII	2009	Nigeria	PLJN -TSI/2009	
		MN046109	II	2017	Nigeria	PLJS-137B /2017	
	**7**	KC689333	XVII	2010	Nigeria	chicken-Nigeria-LC04-2010	(Rinle *et al.*, 2019)
		KC689334	XVII	2010	Nigeria	chicken-Nigeria-LC03-2010	
		KC689335	XVII	2010	Nigeria	chicken-Nigeria-LC5-2010	
	**8**	FM200824	XIV.1	2007		Nigeria/S30/2007	(Snoeck *et al.*, 2009)
		FM200823	XIV.1	2007		Nigeria/S32/2007	
		FM200821	XIV.1	2007		Nigeria/S17/2007	
		FM200820	XVII	2007		Nigeria/S24/2007	
		FM200825	XVII	2007		Nigeria/S36/2007	
		FM200822	XVII	2007		Nigeria/S7/2007	
		FM200796	XVII	2007		fowl/Nigeria/NIE93/2007	
		FM200797	VI.1.1	2007		pigeon/Nigeria/NIE95/2007	
		FM200798	VI.1.1	2007		Parrot/Nigeria/NIE139/2007	
		FM200803	III	2006		chicken/Nigeria/N90/2006	
		FM200799	I	2006		chicken/Nigeria/N400/2006	
		FM200800	I	2005		chicken/Nigeria/SH11/2005	
		FM200801	II	2006		chicken/Nigeria/N2/2006	
		FM200802	II	2006		chicken/Nigeria/N18/2006	
		FM200816	II	2007		turkey/Nigeria/NIE7/2007	
		FM200813	II	2006		chicken/Nigeria/N113/2006	
		FM200814	II	2009		chicken/Nigeria/N239/2009	
		FM200812	II	2006		chicken/Nigeria/N112/2006	
		FM200818	II	2007		Nigeria/S28/2007	
		FM200819	II	2007		Nigeria/S38/2007	
		FM200817	II	2007		turkey/Nigeria/NIE94/2007	
		FM200810	II	2006		chicken/Nigeria/N1/2006	
		FM200811	II	2006		chicken/Nigeria/N106/2006	
		FM200815	II	2007		turkey/Nigeria/NIE6/2007	
	**9**	HQ456869	XVII	2007	Nigeria	APMV-1/duck/Nigeria/Damaturu/26/07	(Solomon *et al.*, 2012)
		HQ456870	XVII	2007		APMV-1/duck/Nigeria/Damaturu/27/07	
		HQ456871	XVII	2007		APMV-1/guinea fowl/Nigeria/Minna/49/07	
		HQ456872	XVII	2007		APMV-1/guinea fowl/Nigeria/Gusau/53/07	
**Togo**	**1**	JX390609	VII	2009	Togo	chicken/Togo/AKO18/2009	(Kim *et al.*, 2012)

	**2**	JX546272	VII	2009	Togo	APMV-1/duck/Togo/GBO5/2009	(Samuel *et al.*, 2013)
		JX546273	VII	2009	Togo	APMV-1/chicken/Togo/AKC12/2009	
		JX546274	VII	2009	Togo	APMV-1/duck/Togo/AKC14/2009	
		JX546275	VII	2009	Togo	APMV-1/chicken/Togo/AKO11/2009	
		JX390609	VII	2009	Togo	APMV-1/chicken/Togo/AKO18/2009	

References

Abah, H. O., Shittu, I., Abdu, P. A., & Aronu, C. J. (2020). Molecular characterization and phylogenetic analysis of Newcastle disease virus isolated from poultry in North Central States of Nigeria. *Journal of Veterinary Medicine and Animal Health*, *12*(2), 20–26. https://doi.org/10.5897/jvmah2019.0825

Cattoli, G., Fusaro, A., Monne, I., Molia, S., Le Menach, A., Maregeya, B., Nchare, A., Bangana, I., Maina, A. G., N'Goran Koffi, J. N., Thiam, H., Bezeid, O. E. M. A., Salviato, A., Nisi, R., Terregino, C., & Capua, I. (2010). Emergence of a new genetic lineage of Newcastle disease virus in West and Central Africa-Implications for diagnosis and control. *Veterinary Microbiology*, *142*(3–4), 168–176. https://doi.org/10.1016/j.vetmic.2009.09.063

da Silva, A. P., Aston, E. J., Chiwanga, G. H., Birakos, A., Muhairwa, A. P., Kayang, B. B., Kelly, T., Zhou, H., & Gallardo, R. A. (2020). Molecular characterization of newcastle disease viruses isolated from chickens in Tanzania and Ghana. *Viruses*, *12*(9). https://doi.org/10.3390/v12090916

de Almeida, R. S., Hammoumi, S., Gil, P., Briand, F. X., Molia, S., Gaidet, N., Cappelle, J., Chevalier, V., Balança, G., Traoré, A., Grillet, C., Maminiaina, O. F., Guendouz, S., Dakouo, M., Samaké, K., Bezeid, O. E. M., Diarra, A., Chaka, H., Goutard, F., … Albina, E. (2013). New Avian Paramyxoviruses Type I Strains Identified in Africa Provide New Outcomes for Phylogeny Reconstruction and Genotype Classification. *PLoS ONE*, *8*(10). https://doi.org/10.1371/journal.pone.0076413

Kim, S.-H., Nayak, S., Paldurai, A., Nayak, B., Samuel, A., Aplogan, G. L., Awoume, K. A., Webby, R. J., Ducatez, M. F., Collins, P. L., & Samal, S. K. (2012). Complete Genome Sequence of a Novel Newcastle Disease Virus Strain Isolated from a Chicken in West Africa. *Journal of Virology*, *86*(20), 11394–11395. https://doi.org/10.1128/jvi.01922-12

Rinle, K. P., Nelson, E. K. A., Said, A. J., Peter, C. T. P., Davou, M. G., Mojisola, A. D., Kelvin, A. S., Gigiya, I. N. D., Blessing, O. S., Joshua, B. I., Rinle, K. P., Nelson, E. K. A., Said, A. J., Peter, C. T. P., Davou, M. G., Mojisola, A. D., Kelvin, A. S., Gigiya, I. N. D., Blessing, O. S., & Joshua, B. I. (2019). Molecular characterization and phylogenetic studies of a virulent newcastle disease virus detected in indigenous chickens in plateau state, Nigeria. *Https://Wjarr.Com/Sites/Default/Files/WJARR-2018-0005.Pdf*, *1*(1), 027–034. https://doi.org/10.30574/WJARR.2019.1.1.0005

Samuel, A., Nayak, B., Paldurai, A., Xiao, S., Aplogan, G. L., Awoume, K. A., Webby, R. J., Ducatez, M. F., Collins, P. L., & Samal, S. K. (2013). Phylogenetic and pathotypic characterization of newcastle disease viruses circulating in west Africa and efficacy of a current vaccine. *Journal of Clinical Microbiology*, *51*(3), 771–781. https://doi.org/10.1128/JCM.02750-12

Snoeck, C. J., Adeyanju, A. T., Owoade, A. A., Couacy-Hymann, E., Alkali, B. R., Ottosson, U., & Muller, C. P. (2013). Genetic diversity of newcastle disease virus in wild birds and pigeons in West Africa. *Applied and Environmental Microbiology*, *79*(24), 7867–7874. https://doi.org/10.1128/AEM.02716-13

Snoeck, C. J., Ducatez, M. F., Owoade, A. A., Faleke, O. O., Alkali, B. R., Tahita, M. C., Tarnagda, Z., Ouedraogo, J. B., Maikano, I., Mbah, P. O., Kremer, J. R., & Muller, C. P. (2009). Newcastle disease virus in West Africa: New virulent strains identified in non-commercial farms. *Archives of Virology*, *154*(1), 47–54. https://doi.org/10.1007/s00705-008-0269-5

Snoeck, C. J., Owoade, A. A., Alkali, B. R., Okwen, M. P., Adeniyi, T., Komoyo, G. F., Nakouné, E., Faou, L., & Muller, C. P. (2013). *High Genetic Diversity of Newcastle Disease Virus in Poultry in West and Central Africa : Cocirculation of Genotype XIV and Newly Defined Genotypes XVII and XVIII*. https://doi.org/10.1128/JCM.00684-13

Snoeck, C. J., Owoade, A. A., Couacy-Hymann, E., Alkali, B. R., Okwen, M. P., Adeyanju, A. T., Komoyo, G. F., Nakouné, E., Le Faou, A., & Muller, C. P. (2013). High genetic diversity of newcastle disease virus in poultry in west and central Africa: Cocirculation of genotype XIV and newly defined genotypes XVII and XVIII. *Journal of Clinical Microbiology*, *51*(7), 2250–2260. https://doi.org/10.1128/JCM.00684-13

Solomon, P., Bisschop, S., Joannis, T. M., Shittu, I., Meseko, C., Sulaiman, L., Gado, D., Oladokun, A. T., Olawuyi, K. A., & Abolnik, C. (2012). Phylogenetic analysis of Newcastle disease viruses isolated from asymptomatic guinea fowls (Numida meleagris) and Muscovy ducks (Cariana moscata) in Nigeria. *Tropical Animal Health and Production*, *45*(1), 53–57. https://doi.org/10.1007/s11250-012-0173-6

Souley, M. M., Issa Ibrahim, A., Souley Kouato, B., Abdou, A., Issa, R., Yaou, B., Amadou, H., Hama, H., Adakal, H., Abdou, N., Cattoli, G., & Dundon, W. G. (2021). Co-circulation of genotypes XIV.2 and XVIII.2 of avian paramyxovirus-1 (Newcastle disease virus) in backyard poultry in Niger. *Virus Genes*, *57*(1), 100–105. https://doi.org/10.1007/s11262-020-01804-x

van Borm, S., Obishakin, E., Joannis, T., Lambrecht, B., & van den Berg, T. (2012). Further evidence for the widespread co-circulation of lineages 4b and 7 velogenic Newcastle disease viruses in rural Nigeria. *Avian Pathology*, *41*(4), 377–382. https://doi.org/10.1080/03079457.2012.696311

Welch, C. N., Shittu, I., Abolnik, C., Solomon, P., Dimitrov, K. M., Taylor, T. L., Williams-Coplin, D., Goraichuk, I. V., Meseko, C. A., Ibu, J. O., Gado, D. A., Joannis, T. M., & Afonso, C. L. (2019). Genomic comparison of Newcastle disease viruses isolated in Nigeria between 2002 and 2015 reveals circulation of highly diverse genotypes and spillover into wild birds. *Archives of Virology*, *164*(8), 2031–2047. https://doi.org/10.1007/s00705-019-

04288-9
