# Supplementary material for: Genomic Diversity and Geographic Distribution of Newcastle Disease Virus Genotypes in Africa: Implications for Diagnosis, Vaccination, and Regional Collaboration
Source: Viruses. 2024 May 16;16(5):795. doi: 10.3390/v16050795 (PMC11125703; doi:10.3390/v16050795)
Supplement: Supplementary file 1 [file viruses-16-00795-s001.zip › Table S4 Distribution of class II NDV genotypes in Central Africa.pdf]

Table S4: Distribution of class II NDV genotypes in Central Africa

Country	Paper	Isolate GenBank accession number	Genotype	Year of collection	Country	Isolate name	Reference
**Cameroon**	**1**	FJ772484	XVII	2008	Cameroon	Cameroon/3490-147/2008	(Cattoli et al., 2010)
		FJ772478	XVII	2008	Cameroon	chicken-3490-149-Cameroon-2008	
	**2**	HF969159	I	2011	Cameroon	chicken/Cameroon/CAE11-855/2011	(Snoeck *et al.*, 2013)
**Central African Republic**	**1**	HF969125	XVII	2008	Central African Republic	chicken/Central African Republic/CAF09-014/2008	(Snoeck *et al.*, 2013)
		HF969181	XVII	2008	Central African Republic	chicken/Central African Republic/CAF09-015/2008	
		HF969182	XVII	2008	Central African Republic	chicken/Central African Republic/CAF09-016/2008	
**Democratic Republic of the Congo**	**1**	MW363929	New variant	2018	Democratic Republic of the Congo	NDV/Ck/DRC/18VIR3696/2018	(Twabela *et al.*, 2021)
		MW363930	New variant	2018	Democratic Republic of the Congo	NDV/Ck/DRC/PL33/2018	
		MW363931	VII.2	2019	Democratic Republic of the Congo	NDV/Ck/DRC/PL219/2019	

References

Cattoli, G., Fusaro, A., Monne, I., Molia, S., Le Menach, A., Maregeya, B., Nchare, A., Bangana, I., Maina, A. G., N'Goran Koffi, J. N., Thiam, H., Bezeid, O. E. M. A., Salviato, A., Nisi, R., Terregino, C., & Capua, I. (2010). Emergence of a new genetic lineage of Newcastle disease virus in West and Central Africa-Implications for diagnosis and control. *Veterinary Microbiology*, *142*(3–4), 168–176. https://doi.org/10.1016/j.vetmic.2009.09.063

Snoeck, C. J., Owoade, A. A., Couacy-Hymann, E., Alkali, B. R., Okwen, M. P., Adeyanju, A. T., Komoyo, G. F., Nakouné, E., Le Faou, A., & Muller, C. P. (2013). High genetic diversity of newcastle disease virus in poultry in west and central Africa: Cocirculation of genotype XIV and newly defined genotypes XVII and XVIII. *Journal of Clinical Microbiology*, *51*(7), 2250–2260. https://doi.org/10.1128/JCM.00684-13

Twabela, A. T., Nguyen, L. T., Masumu, J., Mpoyo, P., Mpiana, S., Sumbu, J., Okamatsu, M., Matsuno, K., Isoda, N., Zecchin, B., Monne, I., & Sakoda, Y. (2021). A new variant among newcastle disease viruses isolated in the democratic republic of the congo in 2018 and 2019. *Viruses*, *13*(2), 1–11. https://doi.org/10.3390/v13020151
