# Supplementary material for: Genomic Diversity and Geographic Distribution of Newcastle Disease Virus Genotypes in Africa: Implications for Diagnosis, Vaccination, and Regional Collaboration
Source: Viruses. 2024 May 16;16(5):795. doi: 10.3390/v16050795 (PMC11125703; doi:10.3390/v16050795)
Supplement: Supplementary file 1 [file viruses-16-00795-s001.zip › Table S5 Distribution of class II NDV genotypes in Northern Africa.pdf]

Table S5: Distribution of class II NDV genotypes in Northern Africa

Country	Paper	Isolate GenBank accession number	Geno type	Year of collection	Country	Isolate name	Reference
**Egypt**	**1 (UNVERIFIED)**	KU377781	VII 1.1	2015	Egypt	NDV/ EG/CH/ 18/2015	(Abd El Aziz *et al.*, 2016)
		KU377782	VII 1.1	2015	Egypt	NDV/EG/CH/24/2014	
		KU377783	VII 1.1	2015	Egypt	NDV/EG/CH/26/2014	
		KU377784	VII 1.1	2015	Egypt	NDV /EG/ CH/ 35/ 2014	
	**2**	FJ939313	II	2005	Egypt	NDV/Chicken/Egypt/1/2005	(Mohamed et al., 2009)
	**3**	JX504077	VII 1.1	2012	Egypt	NDV/Egy/SH03/2012	(Abdel-Glil *et al.*, 2014)
		JX504078	VII 1.1	2012	Egypt	NDV/Egy/SH02/2012	
		JX504079	VII 1.1	2012	Egypt	NDV/Egy/SH04/2012	
		JX504080	VII 1.1	2012	Egypt	NDV/Egy/SH01/2012	
	**4**	MK604197	VII	2015	Egypt	NDV-EGYPT/1516F-2015	(Zanaty *et al.*, 2019)
		MK604198	VII	2015	Egypt	NDV-EGYPT/151309F-2015	
		MK604199	VII	2015	Egypt	NDV-EGYPT/15492F-2015	
		MK604200	VII	2015	Egypt	NDV-EGYPT/15986F-2015	
		MK604201	VII	2015	Egypt	NDV-EGYPT/15158F-2015	
		MK604202	VII	2016	Egypt	NDV-EGYPT/16280F-2016	
		MK604203	VII	2016	Egypt	NDV-EGYPT/16261F-2016	
		MK604204	VII	2016	Egypt	NDV-EGYPT/16553F-2016	
		MK604205	II	2016	Egypt	NDV-EGYPT/1610CV-2016	
		MK604206	VII	2016	Egypt	NDV-EGYPT/161158F-2016	
		MK604207	VII	2017	Egypt	NDV-EGYPT/17295F-2017	
		MK604208	VII	2017	Egypt	NDV-EGYPT/17273F-2017	
		MK604209	VII	2017	Egypt	NDV-EGYPT/171014F-14-2017	
		MK604210	VII	2017	Egypt	NDV-EGYPT/171014F-15-2017	
		MK604211	VII	2017	Egypt	NDV-EGYPT/171014F-16-2017	
		MK604212	II	2017	Egypt	NDV-EGYPT/17140F/S-2017	
		MK604213	II	2017	Egypt	NDV-EGYPT/17151F/S-2017	
		MK604214	II	2018	Egypt	NDV-EGYPT/18156F/S-2018	
		MK604215	I	2018	Egypt	NDV-EGYPT/18246F/S-2018	

		MK604216	VII	2018	Egypt	NDV-EGYPT/181210F-2018	
		MK604217	VII	2018	Egypt	NDV-EGYPT/18734F-2018	
		MK604218	VII	2018	Egypt	NDV-EGYPT/18403F-2018	
		MK604219	VII	2018	Egypt	NDV-EGYPT/18629F-2018	
		MK604220	VII	2018	Egypt	NDV-EGYPT/18238F-2018	
		MK604221	VII	2018	Egypt	NDV-EGYPT/1890FI-2018	
	**5**	KY075880	VII 1.1	2016	Egypt	NDV/chicken/Egypt/Sharkia7/2016	(Orabi *et al.*, 2017)
		KY075881	VII 1.1	2016	Egypt	NDV/chicken/Egypt/Ismailia8/2016	
		KY075882	VII 1.1	2016	Egypt	NDV/chicken/Egypt/Dametta9/2016	
		KY075883	VII 1.1	2016	Egypt	NDV/chicken/Egypt/Sharkia10/2016	
		KY075884	VII 1.1	2016	Egypt	NDV/chicken/Egypt/Qualyobia11/2016	
		KY075885	VII 1.1	2016	Egypt	NDV/chicken/Egypt/Qualyobia12/2016	
		KY075886	VII 1.1	2016	Egypt	NDV/chicken/Egypt/Sharkia14/2016	
		KY075887	VII 1.1	2016	Egypt	NDV/chicken/Egypt/El-Arish15/2016	
		KY075888	VII 1.1	2016	Egypt	NDV/chicken/Egypt/El-Arish16/2016	
		KY075889	VII 1.1	2016	Egypt	NDV/chicken/Egypt/Qualyobia26/2016	
		KY075890	VII 1.1	2016	Egypt	NDV/chicken/Egypt/Dakahlia27/2016	
		KY075891	VII 1.1	2016	Egypt	NDV/chicken/Egypt/Dakahlia28/2016	
		KY075892	VII 1.1	2016	Egypt	NDV/chicken/Egypt/Ismailia29/2016	
		KY075893	VII 1.1	2016	Egypt	NDV/chicken/Egypt/Dakahlia30/2016	
		KY075894	VII 1.1	2016	Egypt	NDV/chicken/Egypt/Dakahlia31/2016	
		KY075895	VII 1.1	2016	Egypt	NDV/chicken/Egypt/Ismailia32/2016	
	**6**	KX231852	VII.1.1	2015	Egypt	NDV/chicken/Egypt/1/2015	(Megahed *et al.*, 2018)
		KX231853	VII.1.1	2015	Egypt	NDV/chicken/Egypt/2/2015	
		KX231851	VII.1.1	2015	Egypt	NDV/chicken/Egypt/3/2015	
		KX231854	VII.1.1	2015	Egypt	NDV/chicken/Egypt/4/2015	
	**7**	MN905162	VII.1.1	2019	Egypt	NDv Giza S4	(Shakal *et al.*, 2020)
	**8**	MF418017	VII	2007	Egypt	NDV/Chicken/EG-MN/NRC/2015	(Ahmed *et al.*, 2017)
		MF418018	VII	2007	Egypt	NDV/Chicken/EG-QU/NRC/2015	
		MF418019	VII	2007	Egypt	NDV/Chicken/EG-SH/NRC/2015	

		MF418020	VII	2007	Egypt	NDV/Chicken/EG -SH2/ NR/2015	
	**9**	MG966505	VII.1.1	2014	Egypt	NDV/CHICKEN/EGYPT/48 Ob/NRC-CU/2014	(Amer, 2018)
		MG966506	VII.1.1	2014	Egypt	NDV/CHICKEN/EGYPT/66 Oc/NRC/2014	
	**10**	MN137989	VII.1.1	2019	Egypt	NDV/EG-Q/Ch/6/2019	(Desouky *et al.*, 2020)
		MN137990	VII.1.1	2018	Egypt	NDV/EG-Q/Ch/31/2018	
		MN137991	VII.1.1	2018	Egypt	NDV/EG-Q/Ch/11/2018	
		MN137992	VII.1.1	2019	Egypt	NDV/EG-Q/Ch/47/2019	
		MN458502	VII.1.1	2019	Egypt	NDV/EG-Q/Ch/4/2019	
		MN458503	VII.1.1	2019	Egypt	NDV/EG-Q/Ch/5/2019	
		MN458504	VII.1.1	2018	Egypt	NDV/EG-Q/Ch/9/2018	
		MN458505	VII.1.1	2018	Egypt	NDV/EG-Q/Ch/12/2018	
	**11**	JX173098	VII.1.1	2012	Egypt	NDV/Chicken/Giza/Egypt/ MR0/2012	(Radwan *et al.*, 2013)
		JX193771	II	2012	Egypt	NDV/Chicken/Behaira/Egy pt/MR6/2012	
		JX193772	II	2011	Egypt	NDV/Chicken/ Fayoum/Egypt/MR7/2011	
	**12**	MHO78055	VII.1.1	2016	Egypt	NDV-ch-EG-CLEVB-F604-2016	(Monir *et al.*, 2018)
	**13**	MT878465	VII.1.1	2017	Egypt	Cattle_egret/Egypt/KFS-Elhamoul-1/2017	(Abd Elfatah *et al.*, 2021)
		MT878466	VII.1.1	2018	Egypt	Cattle_egret/Egypt/KFS-Motobas-2/2018	
		MT878467	VII.1.1	2017	Egypt	Sparrow/Egypt/KFS-Elhamoul-3/2017	
	**14**	MK495878	VII.1.1	2011	Egypt	NDV/Egypt/Luxor/2011/1	(Tran *et al.*, 2020)
		MK495879	VII.1.1	2011	Egypt	NDV/Egypt/Luxor/2011/2	
		MK495883	VII.1.1	2012	Egypt	NDV/Egypt/Luxor/2012/2	
		MK495884	VII.1.1	2012	Egypt	NDV/Egypt/Luxor/2012/3	
		MK495885	VII.1.1	2012	Egypt	NDV/Egypt/Luxor/2012/4	
		MK495886	VII.1.1	2012	Egypt	NDV/Egypt/Luxor/2012/5	
		MK495887	VII.1.1	2012	Egypt	NDV/Egypt/Luxor/2012/7	
		MK495906	VII.1.1	2012	Egypt	NDV/Egypt/Qena/2012/2	
		MK495909	VII.1.1	2013	Egypt	NDV/Egypt/Aswan/2013/ 1	
		MK495910	VII.1.1	2013	Egypt	NDV/Egypt/Luxor/2013/1	

	**15**	KY693681	VII.1.1	2014	Egypt	VNDV/CK/EG/VIR-SHABS/2014	(Ewies *et al.*, 2017)
		KY693682	VII.1.1	2014	Egypt	NDV/CK/EG/VIR-MMBS/2014	
		KY693683	VII.1.1	2016	Egypt	VNDV/CK/EG/VIR-FAY02/2016	
		KY693684	VII.1.1	2016	Egypt	VNDV/CK/EG/VIR-B.S01/2016	
	**16**	AB871426	VII.1.1	2011	Egypt	NDV/EG/CK/13/11	(Sultan *et al.*, 2015)
		AB871425	VII.1.1	2011	Egypt	NDV/EG/CK/20/11	
		AB871428	VII.1.1	2011	Egypt	NDV/EG/CK/67/11	
		AB871422	VII.1.1	2011	Egypt	NDV/EG/CK/83/11	
		AB871427	VII.1.1	2012	Egypt	NDV/EG/CK/101/12	
		AB871423	VII.1.1	2012	Egypt	NDV/EG/CK/129/12	
		AB863742	VII.1.1	2012	Egypt	NDV/EG/CK/135/12	
		AB871424	VII.1.1	2012	Egypt	NDV/EG/CK/146/12	
	**17**	MH445410	VII.1.1	2015	Egypt	NDV-Egypt-18-2015	(Abd El-Hamid *et al.*, 2020)
		MN519688	VII.1.1	2017	Egypt	NDV/Egypt/Ch/MR89/2017	
		MK984236	VII.1.1	2017	Egypt	NDV/Egypt/Ch/B36-F0/2017	
		MK984237	VII.1.1	2018	Egypt	NDV/Egypt/Ch/D30-F0/2018	
		MN519690	VII.1.1	2018	Egypt	NDV/Egypt/Ch/MH6/2018	
		MN519692	VII.1.1	2018	Egypt	NDV/Egypt/Ch/MDK3/2018	
		MN519693	VII.1.1	2018	Egypt	NDV/Egypt/Ch/MQ3/2018	
		MK984238	VII.1.1	2018	Egypt	NDV/Egypt/Ch/R78-F0/2018	
		MN519689	VII.1.1	2018	Egypt	NDV/Egypt/Ch/MGhl/2018	
		MN519694	VII.1.1	2018	Egypt	NDV/Egypt/Ch/MK/2018	
		MN519687	VII.1.1	2018	Egypt	NDV/Egypt/Ch/MQ4/2018	
		MN519685	VII.1.1	2018	Egypt	NDV/Egypt/P/MR82/2018	
		MN519686	VII.1.1	2018	Egypt	NDV/Egypt/P/MR84/2018	
		MN519684	VII.1.1	2019	Egypt	NDV/Egypt/Ch/MN51/2019	
	**18**	FJ969393	II	2006	Egypt	NDV/chicken/Egypt/2/2006	(Mohamed *et al.*, 2011)
		FJ969394	II	2006	Egypt	NDV/chicken/Egypt/3/2006	

		FJ969395	II	2006	Egypt	NDV/chicken/Egypt/4/2006	
	**19**	KM288609	VII	2012	Egypt	NDV-B7-RLQP-CH-EG-12	(Saad *et al.*, 2017)
		KM288610	VII	2012	Egypt	NDV-B23-RLQP-CH-EG-12	
		KM288611	VII	2012	Egypt	NDV-B34-RLQP-CH-EG-12	
		KM288612	VII	2012	Egypt	NDV-B83-RLQP-CH-EG-12	
		KM288613	VII	2012	Egypt	NDV-B102-RLQP-CH-EG-12	
		KM288614	VII	2012	Egypt	NDV-B106-RLQP-CH-EG-12	
		KM288615	VII	2012	Egypt	NDV-B109-RLQP-CH-EG-12	
		KM288616	VII	2012	Egypt	NDV-B110-RLQP-CH-EG-12	
		KM288617	VII	2012	Egypt	NDV-B113-RLQP-CH-EG-12	
		KM288618	VII	2012	Egypt	NDV-B114-RLQP-CH-EG-12	
		KM288619	VII	2012	Egypt	NDV-B127-RLQP-CH-EG-12	
		KM288620	VII	2012	Egypt	NDV-B161-RLQP-CH-EG-12	
		KM288621	VII	2011	Egypt	NDV-F278-RLQP-CH-EG-11	
		KM288622	II	2012	Egypt	NDV-B81-RLQP-CH-EG-12	
		KP316015	VII	2013	Egypt	NDV-F460-RLQP-CH-EG-13	
		KP316016	VII	2014	Egypt	NDV-F388-RLQP-CH-EG-14	
			XXI.1.1	2021	Egypt	Chicken/Egypt/BSU24/2021	(Abozaid & Abdel-Moneim, 2022)
**Libya**	**1**	KP719224	VII.2	2013	Libya	APMV-1/chicken/Libya/13VIR/7225-1/2013	(Kammon *et al.*, 2015)
	**2**		VII.2	2015	Libya	APMV1/Libya/15VIR5368/2015	(Kammon *et al.*, 2018)
			VII.2	2015	Libya	APMV1/Libya/15VIR5371/2015	
			VI.1.1	2015	Libya	APMV1/Libya/15VIR5369/2015	

References

Abd El-Hamid, H. S., Shafi, M. E., Albaqami, N. M., Ellakany, H. F., Abdelaziz, N. M., Abdelaziz, M. N., Abd El-Hack, M. E., Taha, A. E., Alanazi, K. M., & Elbestawy, A. R. (2020). Sequence analysis and pathogenicity of Avian Orthoavulavirus 1 strains isolated from poultry flocks during 2015-2019. *BMC Veterinary Research*, *16*(1), 1–15. https://doi.org/10.1186/s12917-020-02470-9

Abd El Aziz, M., Abd El-Hamid, H., Ellkany, H., Nasef, S., Nasr, S., & El Bestawy, A. (2016). Biological and Molecular Characterization of Newcastle Disease Virus Circulating in Chicken Flocks, Egypt, During 2014-2015. *Journal, Zagazig Veterinary*, *44*(1), 9–20. https://doi.org/10.21608/zvjz.2016.7827.Biological

Abd Elfatah, K. S., Elabasy, M. A., El-Khyate, F., Elmahallawy, E. K., Mosad, S. M., El-Gohary, F. A., Abdo, W., Al-Brakati, A., Seadawy, M. G., Tahoon, A. E., & El-Gohary, A. E. (2021). Molecular characterization of velogenic newcastle disease virus (Sub-genotype vii.1.1) from wild birds, with assessment of its pathogenicity in susceptible chickens. *Animals*, *11*(2), 1–20. https://doi.org/10.3390/ani11020505

Abdel-Glil, M. Y., Mor, S. K., Sharafeldin, T. A., Porter, R. E., & Goyal, S. M. (2014). Detection and characterization of Newcastle disease virus in formalin-fixed, paraffin-embedded tissues from commercial broilers in Egypt. *Avian Diseases*, *58*(1), 118–123. https://doi.org/10.1637/10616-071813-Reg.1

Abozaid, K. G. A., & Abdel-Moneim, A. S. (2022). Epidemiological surveillance of Newcastle disease virus in Egypt — a 6-year cohort study. *Tropical Animal Health and Production*, *54*(4). https://doi.org/10.1007/s11250-022-03234-3

Ahmed, H. M., Amer, M. M., El-bayoumi, K., Amer, S. A., & Kutkat, M. A. E. A. (2017). Identification and sequencing of Genotype VII of Newcastle disease virus from chicken flocks in six Egyptian Governorates. *Egyptian Journal of Veterinary Sciences*, *48*(1), 31–41. https://doi.org/10.21608/EJVS.2017.1236.1015

Amer, M. (2018). Isolation and Identification of H9N2 influenza and Newcastle disease viruses co-infections in chicken. *Egyptian Journal of Veterinary Sciences*, *0*(0), 0–0. https://doi.org/10.21608/ejvs.2018.4963.1043

Desouky, A., Saad, A., Elshorbagy, M., & Abodalal, S. (2020). Isolation and Identification Of New Sub-Genotypes Of Virulent ND Virus In Broiler Chickens In Qalybia Governorate. *Benha Veterinary Medical Journal*, *39*(1), 159–164. https://doi.org/10.21608/bvmj.2020.32932.1216

Ewies, S. S., Ali, A., Tamam, S. M., & Madbouly, H. M. (2017). Molecular characterization of Newcastle disease virus (genotype VII) from broiler chickens in Egypt. *Beni-Suef University Journal of Basic and Applied Sciences*, *6*(3), 232–237. https://doi.org/10.1016/j.bjbas.2017.04.004

Kammon, A., Heidari, A., Dayhum, A., Eldaghayes, I., Sharif, M., Monne, I., Cattoli, G., Asheg, A., Farhat, M., & Kraim, E. (2015). Characterization of Avian Influenza and Newcastle Disease Viruses from Poultry in Libya. *Avian Diseases*, *59*(3), 422–430. https://doi.org/10.1637/11068-032215-ResNote.1

Kammon, A., Monne, I., Asheg, A., & Cattoli, G. (2018). Molecular detection and characterisation of avian paramyxovirus type 1 in backyard chickens and pigeons in Alzintan city of Libya. *Open Veterinary Journal*, *8*(4), 401–405. https://doi.org/10.4314/ovj.v8i4.8

Megahed, M. M., Eid, A. A. M., Mohamed, W., & Hassanin, O. (2018). Genetic characterization of egyptian newcastle disease virus strains isolated from flocks vaccinated against newcastle disease virus, 2014-2015. *Slovenian Veterinary Research*, *55*(Suppl 20), 17–29. https://doi.org/10.26873/SVR-623-2018

Mohamed, M. H. A., Kumar, S., Paldurai, A., Megahed, M. M., Ghanem, I. A., Lebdah, M. A., & Samal, S. K. (2009). Complete genome sequence of a virulent Newcastle disease virus

isolated from an outbreak in chickens in Egypt. *Virus Genes*, *39*(2), 234–237. https://doi.org/10.1007/S11262-009-0385-7

Mohamed, M. H. A., Kumar, S., Paldurai, A., Samal, S. K., MH, M., S, K., A, P., & SK, S. (2011). Sequence analysis of fusion protein gene of Newcastle disease virus isolated from outbreaks in Egypt during 2006. *Virology Journal*, *8*(1), 237. https://doi.org/10.1186/1743-422X-8-237

Monir, N., Omar, L., El-Bagoury, G., Sharawi, S., & A., A. (2018). Isolation and Molecular Identification of Avian Paramyxovirus-1 from Broiler Outbreak in Qaliobia Governorate, Egypt. *Benha Veterinary Medical Journal*, *34*(2), 265–277. https://doi.org/10.21608/bvmj.2018.29437

Orabi, A., Hussein, A., Saleh, A. A., El-Magd, M. A., & Munir, M. (2017). Evolutionary insights into the fusion protein of Newcastle disease virus isolated from vaccinated chickens in 2016 in Egypt. *Archives of Virology*, *162*(10), 3069–3079. https://doi.org/10.1007/s00705-017-3483-1

Radwan, M. M., Darwish, S. F., El-Sabagh, I. M., El-Sanousi, A. A., & Shalaby, M. A. (2013). Isolation and molecular characterization of Newcastle disease virus genotypes II and VIId in Egypt between 2011 and 2012. *Virus Genes*, *47*(2), 311–316. https://doi.org/10.1007/s11262-013-0950-y

Saad, A. M., Samy, A., Soliman, M. A., Arafa, A., Zanaty, A., Hassan, M. K., Sultan, A. H., Bazid, A. I., & Hussein, A. H. (2017). Genotypic and pathogenic characterization of genotype VII Newcastle disease viruses isolated from commercial farms in Egypt and evaluation of heterologous antibody responses. *Archives of Virology*, *162*(7), 1985–1994. https://doi.org/10.1007/s00705-017-3336-y

Shakal, M., Maher, M., Metwally, A. S., AbdelSabour, M. A., Madbbouly, Y. M., & Safwat, G. (2020). Molecular Identification of a Velogenic Newcastle Disease Virus Strain Isolated from Egypt. *Journal of World's Poultry Research*, *10*, 195–205. https://doi.org/10.36380/JWPR.2020.25

Sultan, S., Osman, N., Ahmed, A. I., Ibrahim, R. S., & Sabra, M. (2015). Phylogenetic Characterization of Velogenic Newcastle Disease Viruses Isolated From Field Outbreaks Among Vaccinated Broiler Chickens in Upper Egypt. *Assiut Veterinary Medical Journal*, *61*(145), 126–135. https://doi.org/10.21608/avmj.2015.170195

Tran, G. T. H., Sultan, S., Osman, N., Hassan, M. I., VAN DONG, H., Dao, T. D., Omatsu, T., Katayama, Y., Mizutani, T., Takeda, Y., Ogawa, H., & Imai, K. (2020). Molecular characterization of full genome sequences of newcastle disease viruses circulating among vaccinated chickens in Egypt during 2011–2013. *Journal of Veterinary Medical Science*, *82*(6), 809–816. https://doi.org/10.1292/jvms.19-0623

Zanaty, A. M., Hagag, N. M., Rabie, N., Selim, M. S. K., Mousa, S. A., Shalaby, A. G., Arafa, A.-S., & Hassan, M. K. (2019). Epidemiological, Phylogenetic Analysis and Pathogenicity of Newcastle Disease Virus Circulating in Poultry Farms, Egypt during 2015-2018. *Hosts and Viruses*, *6*(3). https://doi.org/10.17582/journal.hv/2019/6.3.50.59
